# Altered Genome-Wide DNA Methylation in the Duodenum of Common Variable Immunodeficiency Patients

**DOI:** 10.1007/s10875-024-01726-5

**Published:** 2024-05-23

**Authors:** Mingyi Yang, Mari Kaarbø, Vegard Myhre, Henrik M. Reims, Tom H. Karlsen, Junbai Wang, Torbjørn Rognes, Bente Halvorsen, Børre Fevang, Knut E. A. Lundin, Pål Aukrust, Magnar Bjørås, Silje F. Jørgensen

**Affiliations:** 1 Department of Microbiology, Oslo University Hospital and University of Oslo, Oslo, Norway; 2 Department of Medical Biochemistry, Oslo University Hospital and University of Oslo, Oslo, Norway; 3 Research Institute of Internal Medicine, Division of Surgery, Inflammatory Diseases and Transplantation, Oslo University Hospital, Oslo, Norway; 4 Institute of Clinical Medicine, University of Oslo, Oslo, Norway; 5 Department of Pathology, Oslo University Hospital, Rikshospitalet, Oslo Norway; 6 Norwegian PSC Research Center, Department of Transplantation Medicine, Oslo University Hospital, Oslo, Norway; 7 Section of Gastroenterology, Department of Transplantation Medicine, Oslo University Hospital, Rikshospitalet, Oslo Norway; 8 Department of Clinical Molecular Biology (EpiGen), Akershus University Hospital and University of Oslo, Lørenskog, Norway; 9 Centre for Bioinformatics, Department of Informatics, University of Oslo, Oslo, Norway; 10 Section of Clinical Immunology and Infectious Diseases, Oslo University Hospital, Rikshospitalet, Oslo Norway; 11 Department of Clinical and Molecular Medicine, Norwegian University of Science and Technology, NTNU, Trondheim, Norway; 12 The Proteomics and Modomics Experimental Core Facility (PROMEC) at Norwegian University of Science and Technology, Trondheim, Norway

**Keywords:** CVID, Gastrointestinal tract, Duodenum, Celiac disease, Primary immunodeficiency, Epigenetic, 5-methylcytosine, Genome-wide DNA methylation, CpG regions, TNF

## Abstract

**Purpose:**

A large proportion of Common variable immunodeficiency (CVID) patients has duodenal inflammation with increased intraepithelial lymphocytes (IEL) of unknown aetiology. The histologic similarities to celiac disease, lead to confusion regarding treatment (gluten-free diet) of these patients. We aimed to elucidate the role of epigenetic DNA methylation in the aetiology of duodenal inflammation in CVID and differentiate it from true celiac disease.

**Methods:**

DNA was isolated from snap-frozen pieces of duodenal biopsies and analysed for differences in genome-wide epigenetic DNA methylation between CVID patients with increased IEL (CVID_IEL; n = 5) without IEL (CVID_N; n = 3), celiac disease (n = 3) and healthy controls (n = 3).

**Results:**

The DNA methylation data of 5-methylcytosine in CpG sites separated CVID and celiac diseases from healthy controls. Differential methylation in promoters of genes were identified as potential novel mediators in CVID and celiac disease. There was limited overlap of methylation associated genes between CVID_IEL and Celiac disease. High frequency of differentially methylated CpG sites was detected in over 100 genes nearby transcription start site (TSS) in both CVID_IEL and celiac disease, compared to healthy controls. Differential methylation of genes involved in regulation of TNF/cytokine production were enriched in CVID_IEL, compared to healthy controls.

**Conclusion:**

This is the first study to reveal a role of epigenetic DNA methylation in the etiology of duodenal inflammation of CVID patients, distinguishing CVID_IEL from celiac disease. We identified potential biomarkers and therapeutic targets within gene promotors and in high-frequency differentially methylated CpG regions proximal to TSS in both CVID_IEL and celiac disease.

**Supplementary Information:**

The online version contains supplementary material available at 10.1007/s10875-024-01726-5.

## Introduction

Common variable immunodeficiency (CVID) is a rare disease, but still the most common symptomatic primary immunodeficiency in adults, with a prevalence of 1:25,000 to 1:50,000 in White people [1]. The immunodeficiency is characterized by a B-cell defect that leads to hypogammaglobulinemia that pre-dispose to recurrent airway infections. In addition, a large subgroup of patients, ~ 70%, has various inflammatory and autoimmune complications, involving T cell and monocyte/macrophage pathology, e.g., immune-mediated cytopenia, inflammatory interstitial lung disease, lymphoid hyperplasia, and enteropathy [2–4]. Importantly, CVID patients with these complications have increased mortality and morbidity compared to other CVID patients, who have normal life expectancy [5].

We have previously shown that gastrointestinal (GI) inflammation and gut microbial changes are associated with systemic inflammation and disease severity in CVID, and the most prevalent site of GI inflammation in CVID is in the proximal part of duodenum [6, 7]. The unknown aetiology of duodenal inflammation, and thereby the lack of significant disease biomarkers as well as effective prevention and treatment options, illustrate some of the key challenges in the field of CVID. Histologically it resembles celiac disease, with increased intraepithelial lymphocytes (IEL), but we have recently shown that the transcriptome in duodenal biopsies was different in CVID with increased IEL as compared to untreated celiac disease. In addition, CVID patients with increased IEL displayed alterations in transcription of genes involved in response to viral infections compared to the other CVID patients [8]. This might suggest that exposure to viruses, and not gluten, contributes to duodenal inflammation in CVID patients. Moreover, there were large differences in protein regulation between CVID with increased IEL compared to celiac disease implying that these are different disease entities [8]. However, like celiac disease, CVID patients with increased IEL also had altered type I interferon (IFN) response [8, 9], and the pathogenesis of the duodenal inflammation in CVID is still not clear. We hypothesize that these pathogenic changes could involve epigenetic modifications.

The rationale for exploring epigenetics in CVID is that monogenic defects are found in 10–30% of patients and the onset of disease can be at any age [10, 11]. This suggests that environmental triggers in genetically susceptible individuals could be of importance, where epigenetic modifications may be involved in the transition from healthy to diseased. However, so far, there are only three previous publications on epigenetics of genome-wide DNA methylation in CVID and all these have reported epigenetic modifications in peripheral B -cells in CVID [12–14]. The GI tract could serve as a crucial intersection between gut immunity and environmental factors including gut microbiota, where epigenetic modifications could be involved in the onset of duodenal inflammation. There are no previous publications on epigenetics in gut biopsies in CVID.

Epigenetic DNA methylation provides the most stable epigenetic mark that plays a significant role in genome regulation, representing a bridge between environmental factors and regulation of gene expression and chromatin structure [15]. DNA methylation involves the transfer of a methyl group to the cytosine, usually followed by guanine residues (CpG methylation) to form 5-methylcytosine (5mC). These modifications affect *when* or *if* a given gene is expressed [15]. Hence, DNA methylation profiling could be key in understanding how epigenetics contributes to gene regulation in disorders like CVID [16].

In this study, we analysed epigenetic modifications (CpG methylation) of the gut mucosa of CVID patients both with and without duodenal inflammation compared to healthy controls, with the aim of uncovering potential new mechanisms of disease in CVID. Moreover, due to the histological resemblances between CVID patients with duodenal inflammation and celiac disease, we also compared the epigenetic regulation between these two conditions.

## Methods

### Ethics

The study was approved by the Regional Committee for Medical and Health Research Ethics and conforms to the principles outlined in the Declaration of Helsinki. Written informed consent was obtained from all participants.

### Study design

The study included an upper endoscopy (GIFHQ190, Olympus, Hamburg, Germany). Intestinal biopsies were collected according to protocol [6] at the Section for Gastroenterological Endoscopy at Oslo University Hospital, Rikshospitalet, Oslo, Norway between 2012–2013. The objective of the study was to compare epigenetic DNA methylation of CpG sites in duodenal biopsy samples from patients with CVID (with and without GI inflammation), untreated celiac disease (histologically similar duodenal inflammation as CVID patients), and healthy controls. The duodenal biopsy samples from the CVID patients were sub-grouped according to the presence of increased IEL (CVID_IEL), or CVID patients with no inflammation (normal) (CVID_N), as previously described [6]. Extended Methods are available in Supplementary information.

CVID was defined as decreased serum levels of IgG, IgA, and/or IgM by a minimum of two standard deviations below the mean for age, while excluding other causes of hypogammaglobulinemia [17]. CVID subgroups were classified as “complications” (i.e., presence of one or more of the following complications: enteropathy, splenomegaly, lymphoid hyperplasia, organ-specific autoimmunity, autoimmune cytopenia, granulomas, lymphoid interstitial pneumonitis, nodular regenerative hyperplasia, or lymphoma) or as “infection only” (i.e., only recurrent bacterial infections in the respiratory tract and absence of the above mentioned complications), based on previously defined criteria [18]. With one exception: CVID enteropathy diagnosis was defined as persistent diarrhea (> 3 months) after exclusion of gastrointestinal infection. It was based on GI symptom questionnaire that was collected at the same time as the biopsies, as part of a cross sectional study [6].

All the eight CVID patients that were included in the present study have been tested for monogenic mutation after the onset of this study. The samples were tested using WGS and a set gene panel consisting of 598 genes known to be associated with primary immunodeficiency (PID) and/or hematological diseases. The list of the genes is available Genetikkportalen (https://www.genetikkportalen.no/find-ngs/1100), version 4.

### Sample preparation for whole genome bisulfite sequencing (WGBS)

DNA was isolated from snap frozen biopsies of duodenal biopsies (n = 3–5) using DNA, RNA and protein Allprep kit (Qiagen, Hilden, Germany) according to the manufacturer’s instructions with a few modifications (Supplementary information).

### Whole genome bisulfite sequencing (WGBS) analysis

The initial processing data of genome-wide 5mC counts was generated by Admera Health (Supplementary information). Additional steps of the WGBS data analysis are described in Supplementary Figure S1 and Supplementary information.

### Statistical analysis

Statistical analyses of t-test and Wilcox rank sum test were performed in R (v4.2.2). Differentially methylated cytosines, promoter and regions in tiled windows were identified using a logistic regression- based test in methylKit package, which modeled the log odds ratio of CpG methylation proportions and tested if treatment (or disease condition) vector had any effect on the outcome of variable using Chi-square test. The p-value was adjusted to q-value using SLIM (Sliding Linear Model) method in controlling false discovery rate (FDR). In GO term enrichment analysis, Fisher´s exact test was performed under clusterProfiler package. The p-value was corrected to p-adjusted by Benjamini–Hochberg procedure. To explore the cell subsets of tissue sample in the methylation sequencing data, we performed deconvolution analysis (in-silico analysis). The cell type was predicted by using a R package granulator with a linear model of qprogwc (quadratic programming non-negative least-squares constraints) [19] utilizing the corresponding bulk RNA-seq dat. The granulator package was downloaded via Bioconductor(Pfister S, Kuettel V, Ferrero E [2023]. granulator: Rapid benchmarking of methods for *in silico* deconvolution of bulk RNA-seq data. 10.18129/B9.bioc.granulator, R package version 1.10.0, https://bioconductor.org/packages/granulator). The reference biomarker data in this package consists of transcription level in 1200 genes and 17 cell types based on single cells RNA-seq. In this package, the biomarkers were limited to immune cells, the signature matrices of sigMatrix_ABIS_S0 were used as reference biomarkers.

## Results

### Clinical characteristics

**Fig. 1:**
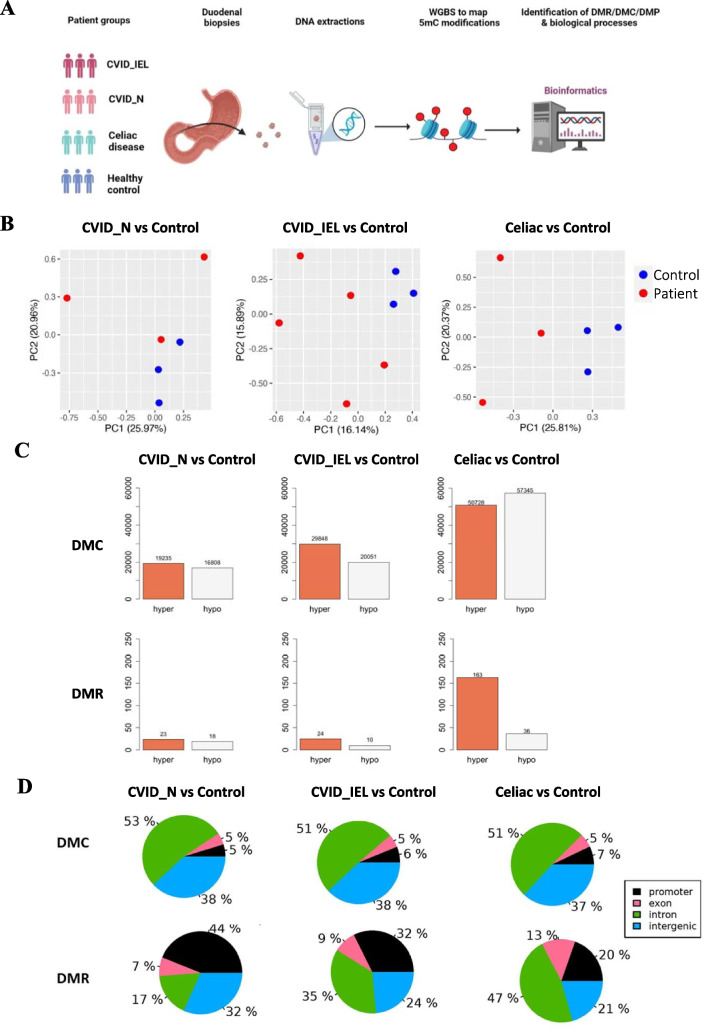
**(A)** Study overview. Duodenal biopsies were obtained by upper endoscopy from patients with Common variable immunodeficiency (CVID) sub-grouped to those with normal duodenal biopsies (CVID_N) or increased intraepithelial lymphocytes (CVID_IEL), healthy controls and celiac disease (also with increased IEL). DNA was extracted from all biopsies. Genome-wide analysis of to map CpG methylation was performed. Differentially methylated cytosines at single base resolution (DMC), in CpG region (DMR) or in promoter region (DMP) were identified between groups, and their relation to biological processes were explored. **(B)** PCA plots* showing the sample clustering for CVID subgroups and celiac disease compared to healthy controls. The groups are indicated in different colors: blue for control, red for patients. **(C)** Counts of differential methylation at single bases (DMCs) and regions (DMRs). Differential methylation was identified in two categories: single base resolution (DMC) and tiled window with a bin size of 1 kb (DMR). **(D)** Pie plots showing proportion of differential methylation in gene feature regions. * **PCA** plot showing the clustering of samples based on the first and the second most important components. The genome-wide CpG methylation percent data was centralized and the most import variance among samples was calculated by a linear model using the function of PCA Samples in methylKit. The x- and y- axis represent the first and the second component, which captured the most and the second most important variability in the data, respectively. Each point in the plot represents a single sample. The distance between points represents how compositional different the samples are from one another e.g. the more similar the samples are, the closer they are to each other, and likewise the more different the samples are, the further away from each other (judged by both the distance on the X- and y axis). PCA, The Principal Component Analysis; Hypo, Hypo- methylation; hyper, hyper- methylation

A simplified illustration of the study is shown in Fig. 1A. The study cohort consisted of duodenal biopsies from five CVID patients with duodenal inflammation i.e. with increased IEL (CVID_IEL), three CVID patients with normal duodenal biopsies (CVID_N), three with untreated celiac disease (Marsh grade 3a-c) and three healthy controls (Table 1). Celiac disease patients were younger than controls but reflect the normal age of onset of celiac disease. The clinical and immunological features for the CVID cohorts are given in Table 2. Importantly, none of the patients were on any anti-inflammatory or immunomodulating therapy except for immunoglobulin replacement therapy. One CVID patient had a monogenic mutation in TNFAIP3 (first reported in CVID in 2019 [20, 21]). The patient had a classical CVID phenotype with recurrent airway infections with capsulated bacteria and non-infectious complications such as splenomegaly, autoimmune cytopenia and enteropathy, as did several of the other patients (Table 2). The other seven CVID patient did not have any mutation identified with our most recent PID panel.

**Table 1:** Patients’ characteristics

Characteristics	CVID_IEL (*n*=5)	CVID_N (*n*=3)	Controls (*n*=3)	Celiac disease (*n*=3)
Female, *n*	4	1	3	2
Age, mean (range)	45, (35–58)	54, (38–62)	58, (40–67)	27, (25–29)

**Table 2:** The clinical, immunological and genetic phenotype of the CVID cohorts

Phenotypes	CVID_IEL (*n*=5)	CVID_N (*n*=3)	CVID total (*n*=8)
Clinical			
Infection only, n	0	0	0%^a^
Complications^b^	5	3	100% ^a^
*Enteropathy*	5	0	63% ^a^
*Splenomegaly*	2	2	50% ^a^
*Lymphoid hyperplasia*	1	2	38% ^a^
*Organ-specific autoimmunity*	2	0	25% ^a^
*Autoimmune cytopenia*	1	1	25% ^a^
IgG replacement therapy	5	3	100% ^a^
Immunomodulatory treatment	0	0	0% ^a^
Immunological			
Ig at diagnosis			
*Reduced IgA (< 0.7 g/L)*	5	3	100% ^a^
*Reduced IgM (< 0.4 g/L)*	4	3	88% ^a^
*IgG, g/L*^*c*^	2.5 (1.2–3.9)	1.7 (0.5–3.0)	2.5 (0.5–3.9)
B cell sub-classes^d^			
*CD19*^*+*^ *cells of lymphocytes> 1%*	5	3	100% ^a^
*Switched memory B-cells* ≤ *2%*	4	2	75% ^a^
*Transitional B cells <9 %*	3	3	75% ^a^
*CD21*_*low*_ *B cells* ≥ 10%	3	3	75% ^a^
Total B cells (× 10^6^/L) ^c^	72 (31–306)	349 (137–579)	218 (31–579)
Total T cells (× 10^6^/L) ^c^	824 (471–1404)	1730 (1131–3483)	1246 (471–3483)
CD4^+^ T cells (× 10^6^/L) ^c^	496 (170–879)	803 (647–1054)	601 (170–1054)
CD8^+^ T cells (× 10^6^/L) ^c^	426 (303–805)	1008 (301–2429)	430 (301–2429)
NK cells (× 10^6^/L) ^c^	147 (28–362)	129 (101–531)	138 (28–531)
Genetics			
*Known monogenic PID mutation* ^*e*^	1	0	13% ^a^

^a^Percentage of the total CVID Cohort (n=8) that have the phenotype in the first column

^b^None of the patients had granulomas, lymphoid interstitial pneumonitis, nodular regenerative hyperplasia of the liver or lymphoma

^c^Median (minimum-maximum)

^d^B cells are classified according to EURO classification [29]

^e^Whole exome sequencing and then gene panel (covering 598 genes, see methods)

*CVID*, Common variable immunodeficiency; *CVID_IEL*, Subclassification of duodenal biopsies from the CVID patient with increased intra epithelial lymphocytes; *CVID_N*, Subclassification of duodenal biopsies from the CVID patient with no increased intra epithelial lymphocytes (normal); *Ig*, Immunoglobulin; *NK*, natural killer cells; *PID*, primary immunodeficiency

### Genome-wide CpG methylation analysis separates disease phenotypes

The average genome coverage ranged from 8.8 × to 15.2 × per sample. The CpG coverage distribution were similar among samples and groups, showing the highest frequency at about 10 reads per CpG (Supplementary Figure S2). The profile of genome wide 5mC frequency distribution is also similar among samples and displays a peak at about 95–100% and a relatively smaller peak at 0–5% (Supplementary Figure S3).

More than seven million CpG sites (including methylated and non-methylated) were detected in each sample: specifically, 7908606, 9785398 and 11793210 CpG sites were detected in CVID_N, CVID_IEL and Celiac disease, respectively, when each of them was compared to healthy control by differential methylation analysis. PCA analysis of these genome-wide CpG methylation data revealed that the samples were separated by disease group for CVID_N, CVID_IEL and celiac disease when compared to healthy control by (Fig. 1B).

### High degree of differential CpG methylation at single base resolution in CVID and celiac disease compared to healthy controls

Differential 5mC methylation was identified in three categories: (i) CpG methylation at **single base resolution (DMC)**, (ii) differentially methylated **CpG regions (DMR),** and (iii) differentially methylated CpGs at **promoter sites (DMP)**. A large set of DMC (more than 30,000 CpG sites involving more than 9,000 associated genes) was identified in CVID and celiac patients compared to healthy controls, with the largest counts of DMC in celiac and the lowest counts in CVID *without* duodenal inflammation (CVID_N) (Fig. 1C). For the CpG methylation distribution across the genome, we plotted the percentage of differentially methylated bases or regions overlapping with gene feature regions, including exon, intron, promoter and intergenic sites. The proportion pattern in **DMC** was similar for all three comparisons and most frequent in introns (Fig. 1D). Interestingly, the analyses of differentially methylated regions (DMR) demonstrated different patterns across gene feature regions, in which CVID_N versus healthy control revealed the highest degree of differential methylation in the promotor region. CVID_IEL and celiac disease displayed more differential methylation in the intergenic region compared to healthy controls (Fig. 1D). Furthermore, hypo- and hyper-methylation, potentially reflecting activation and suppression of the actual genes(15), were not evenly distributed among chromosomes (Supplementary Figure S4). In CVID patients *without* duodenal inflammation (CVID_N), we observed a higher presence of abnormal hypomethylation of DMRs on chromosome 20 compared to healthy individuals. On the other hand, in CVID patients *with* duodenal inflammation (CVID_IEL) there was a higher occurrence of hypomethylation of DMRs on chromosome 21 compared to healthy controls. This suggests that the patterns of aberrant hypomethylation differ between CVID_N and CVID_IEL patients compared to healthy individuals (see Figure S4 for visual representation of the data). Moreover, this may explain why these two CVID patient groups show differential transcription patterns of certain factors [8].

### Differential methylation in promoters of genes as potential novel mediators in CVID and celiac disease

**Fig. 2:**
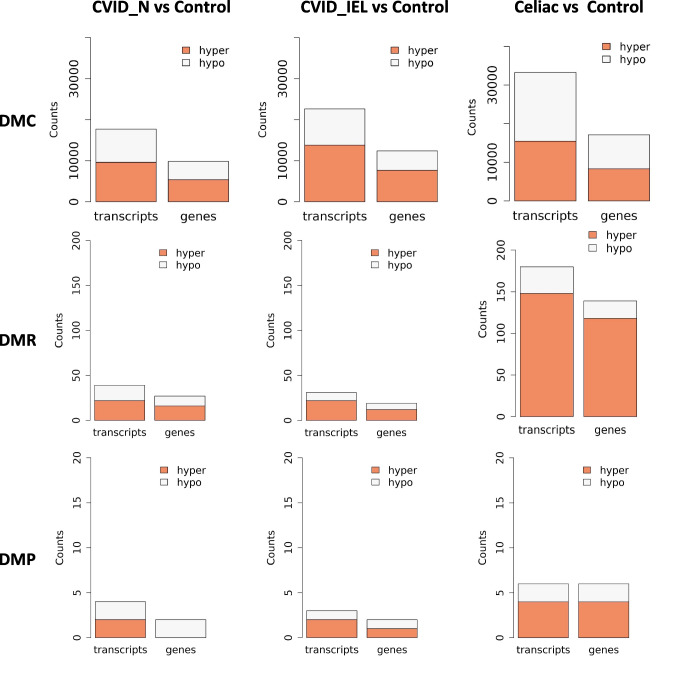
**Bar plots showing the number of associated transcripts and genes in hyper- and hypo-methylated sites**. The differential methylation was identified in three categories: base resolution (DMC), tiled window (DMR) with a bin size of 1 kb and promoter by TSS ± 1 kb (DMP). Each DMC or DMR was annotated with respect to its proximity to a nearby transcript. CVID: Common variable immunodeficiency; Control: healthy controls with normal duodenal biopsies; Celiac: celiac disease Marsh grade 3a-c, CVID_N: CVID patients with normal duodenal biopsies; CVID_IEL: CVID patients with duodenal inflammation.

**Fig. 3:**
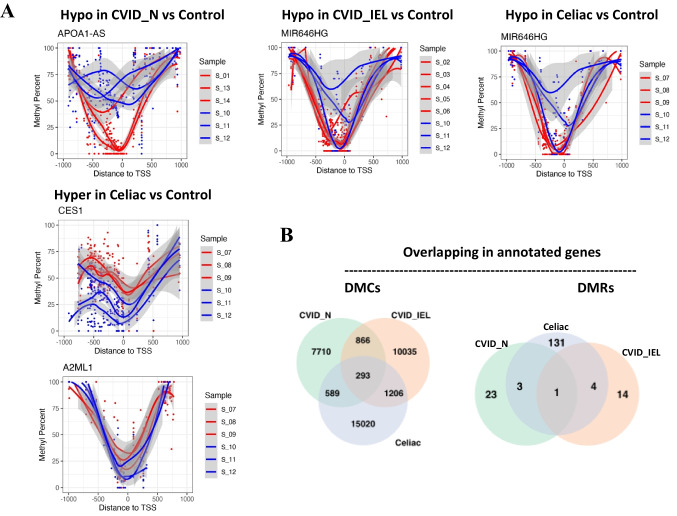
**Visualization of the methylation profile in promoter regions of identified DMP genes. (A)** The promoters of the respective genes that are hypo- or hypermethylated in CVID_N, CVID_IEL, and celiac disease compared to Control. The replicates of the control group labeled in blue and patient groups are labeled in red. **(B)** Venn diagram showing common genes associated with differential methylation (in DMC and DMR) in CVID_IEL, CVID_N, and celiac disease compared to healthy controls. Of note, no common genes were identified in DMP (not shown here). In the diagram, the green circle represents the differentially methylated genes in CVID_N compared to Control, the light orange circle represents CVID_IEL compared to Control, and the blue circle represents celiac disease compared to Control. CVID: Common variable immunodeficiency; Celiac: celiac disease Marsh grade 3 a-c; CVID_N: CVID patients with normal duodenal biopsies; CVID_IEL: CVID patients with duodenal inflammation; DMCs: differential methylations at single base resolution; DMP: differentially methylated promoter region; DMRs: differential methylated regions; TSS: transcription start site.

After exploring the differentially methylated CpG sites and regions, the nearest transcript and genes (with the shortest distance between differential methylation entity and transcription start site [TSS] in genome) were extracted from the Ensembl genome annotation (https://www.ensembl.org). Figure 2 shows the count of differential methylation nearest (associated) transcripts and genes for each paired comparison. In general, celiac patients showed the highest count in the three categories of DMC, DMR and DMP compared to healthy controls. The analyses revealed a high number of DMC associated genes compared to healthy controls in CVID_N (n = 9837), CVID_IEL (n = 12,093) and patients with celiac disease (n = 16,668) (Fig. 2, Supplementary data 1). In contrast, the number of DMP genes compared to controls were low in CVID_N (n = 2), CVID_IEL (n = 2) and celiac disease (n = 6) (Fig. 2, Supplementary data 2). Interestingly, when comparing DMP of CVID_N to controls, the promoter region of *APOA1-AS (APOA1 antisense RNA)* was differently methylated, showing identically hypo-methylated sites in all three replicates of CVID_N compared to healthy controls (Fig. 3A, Supplementary data 2). The promoter of *MIR646HG* was hypo-methylated for both celiac disease and CVID_IEL compared to controls (P < 0.001), but not in CVID_N (Fig. 3A, Supplementary data 2). In addition, the promoter regions of Carboxylesterase 1 (*CES1*), alpha-2-macroglobulin like 1 (A2ML1) and Solute Carrier Family 39 Member 5 (SLC39A5) were significantly hypermethylated in celiac disease (Fig. 3A, Supplementary data 2). Similar to DMP, the number of DMR genes was also relatively low, with only 27, 19 and 139 genes identified in CVID_N, CVID_IEL and celiac disease, respectively (Fig. 2 and Supplementary data 3).

### Little overlap for DMC associated genes among CVID and celiac disease compared to healthy controls

Venn diagram analysis showed that only a small proportion of DMC associated genes (about 10–20%) was overlapping among the three disease categories compared to controls (Fig. 3B). There were 293 associated genes in DMC for the three genotypes compared to controls (Fig. 3B, Supplementary data 4). However, the only common DMR associated gene between CVID_N, CVID_IEL and celiac disease compared to controls, was *SLC6A19* (location in chr5 between position 1,203,001 and 1,204,000, mean methylation difference 28.9%, 1408 base pairs away from the transcription start site) (Supplementary data 4). No common modified genes were found in the DMP among the different groups. When comparing each patient group to the control group, a total of 23, 14 and 131 unique genes were identified in the CVID_N, CVID_IEL and Celiac group, respectively, through gene overlapping analysis of DMRs (Fig. 3B, supplementary data 5). These unique DMR genes in each category have the potential to serve as biomarkers for distinguishing between the CVID subgroups and differentiating CVID_IEL from celiac disease.

### A subset of genes displayed high-frequency DMCs nearby TSS

**Fig. 4:**
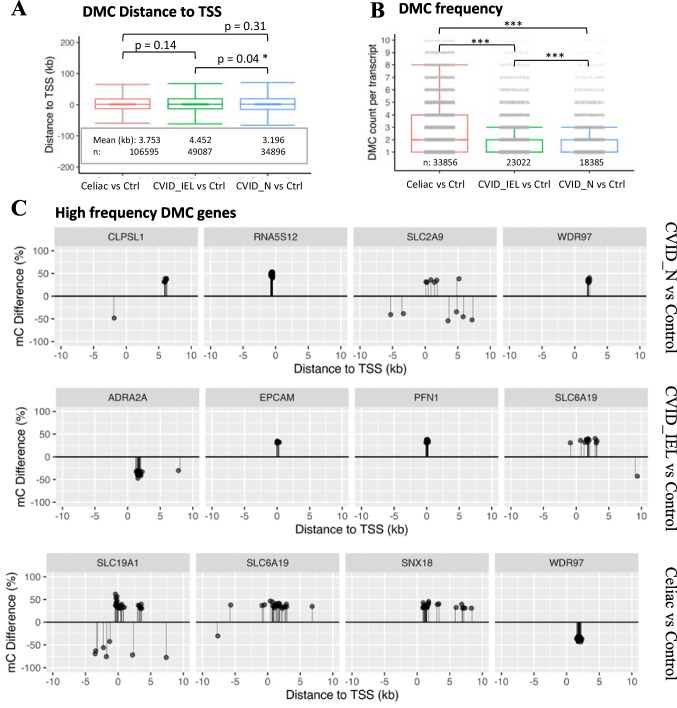
**The distribution pattern of differentially methylated cytosines (DMCs) around transcription start sites (TSS).** (**A)** Boxplot showing the distance from TSS site in all DMCs. Results are given as boxes representing the 25th to 75th percentile with lines indicating median, the whiskers outside the boxes representing the distance of 1.5 times the interquartile range (IQR); the outliers are omitted. *p < 0.05, using t-test between groups (**B)** The distribution of DMC frequency in associated transcripts. All the data are presented as grey points forming parallel grey bars. The higher the density of the bar, the greater number of transcripts included in that bar. The boxes representing the 25th to 75th percentile with lines indicating median (the median values for the green and blue are at the very bottom of the box underneath the dense grey bar). Whiskers over the box indicated the distance of 1.5 times the IQR; The outliers at larger than 10 counts are omitted. ***p < 0.001, using Wilcoxon rank sum test between groups. **(C)** Visualization of the top four genes with high-frequency DMCs in TSS ± 10 kb region. The dot-line represents the position of each differentially methylated CpG (5mC) around TSS. The dot-lines above the x-axis represent the hyper, while those under the x-axis represent the hypo methylation CpGs in patients compared to healthy control. CVID: Common variable immunodeficiency; Celiac: celiac disease Marsh grade 3 a-c; CVID_N: CVID patients with normal duodenal biopsies; CVID_IEL: CVID patients with duodenal inflammation; Ctrl: Control, DMCs: differential methylations at single base resolution; DMRs: differential methylated regions; TSS: transcription start site

To examine the methylation specificity in genes of the patient groups compared to controls, we explored the distribution pattern of differential methylation along the genome. As the count of DMC was much higher than that of DMR and DMP, we focused on DMC in genes. First, we mapped the distance of all DMCs from correlated TSS. We found the majority of DMCs were close to TSS with distance less than 10 kb, the median was close to or at TSS (Fig. 4A, Supplementary Figure S5A). The same pattern was observed for the three patient groups (CVID_N, CVID_IEL and celiac disease). Based on the distance pattern, we further filtered the top DMC associated genes (top DMC genes) by setting the distance to TSS less than 10 kb and the methylation difference larger than 30%. Total 3166–5280 top DMC genes were extracted in each of paired group comparison (Supplementary data 6, top DMC associated genes). The subset of top DMC genes was used to explore their impact on biology by gene ontology (GO) enrichment analysis.

**Fig. 5:**
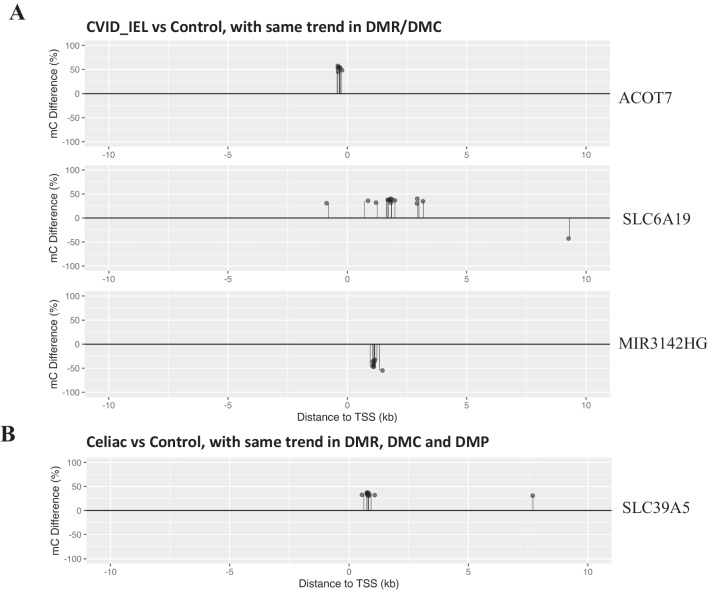
**Visualization of the high-frequency DMCs in TSS ± 10 kb region of genes identified with the same trend in DMR and/or in DMP.** (**A)** ACOT7 and SLC6A19 were hypermethylated in DMCs in CVID_IEL patients compared to healthy controls and were also identified in the DMR of CVID_IEL patients. MIR3142HG was hypomethylated in DMR of CVID_IEL patients. (**B)** SLC39A5 was hypermethylated in DMR and DMP of Celiac patients. CVID: Common variable immunodeficiency; Celiac: celiac disease Marsh grade 3 a-c; CVID_N: CVID patients with normal duodenal biopsies; CVID_IEL: CVID patients with duodenal inflammation; DMCs: differential methylations at single base resolution; DMRs: differential methylated regions; differential methylated at promoter sites (DMP); TSS: transcription start site

To detect genes with high-frequency DMCs and visualize their distribution, DMCs were quantified per transcript within the TSS ± 10 kb region. On average, each transcript displayed one to three DMCs within the analysed TSS ± 10 kb region. The median value of DMC counts per transcript was higher in celiac disease compared to CVID_IEL (P < 0.001), and CVID_N (P < 0.001, Fig. 4B, Supplementary Figure S5B). Notably, a subset of targeting transcripts carried three to ten DMCs or more. Based on the frequency patterns, we defined the high-frequency DMC gene by setting the frequency to at least five DMCs per transcript. We identified a total number of 32, 125 and 736 high-frequency genes in CVID_N, CVID_IEL and celiac disease, respectively, compared to control (Supplementary data 7). All of them carried five to 26 DMCs per gene in TSS ± 10 kb region. The top four genes ranked by DMCs count are shown in Fig. 4C. Next, we examined and visualized the overlapping genes identified in both DMR and high-frequency DMC. Among the high-frequency DMC genes in CVID_IEL patients, the *ACOT7, SLC6A19* in the hypermethylated group and *MIR3142HG* in the hypomethylated group, were also found to exhibit the same pattern of differential methylation (DMR) analysis (Fig. 5A). Similarly, among the high-frequency DMC genes in celiac patients, there were 35 common genes that also overlapped with DMR. Remarkably, *SLC39A5*, overlapped with DMP, showing a clustering of high density hyper DMCs in the region around TSS + 1 kb (Fig. 5B).

### Top DMC genes in relation to biological processes in patient groups compared to healthy controls

**Fig. 6:**
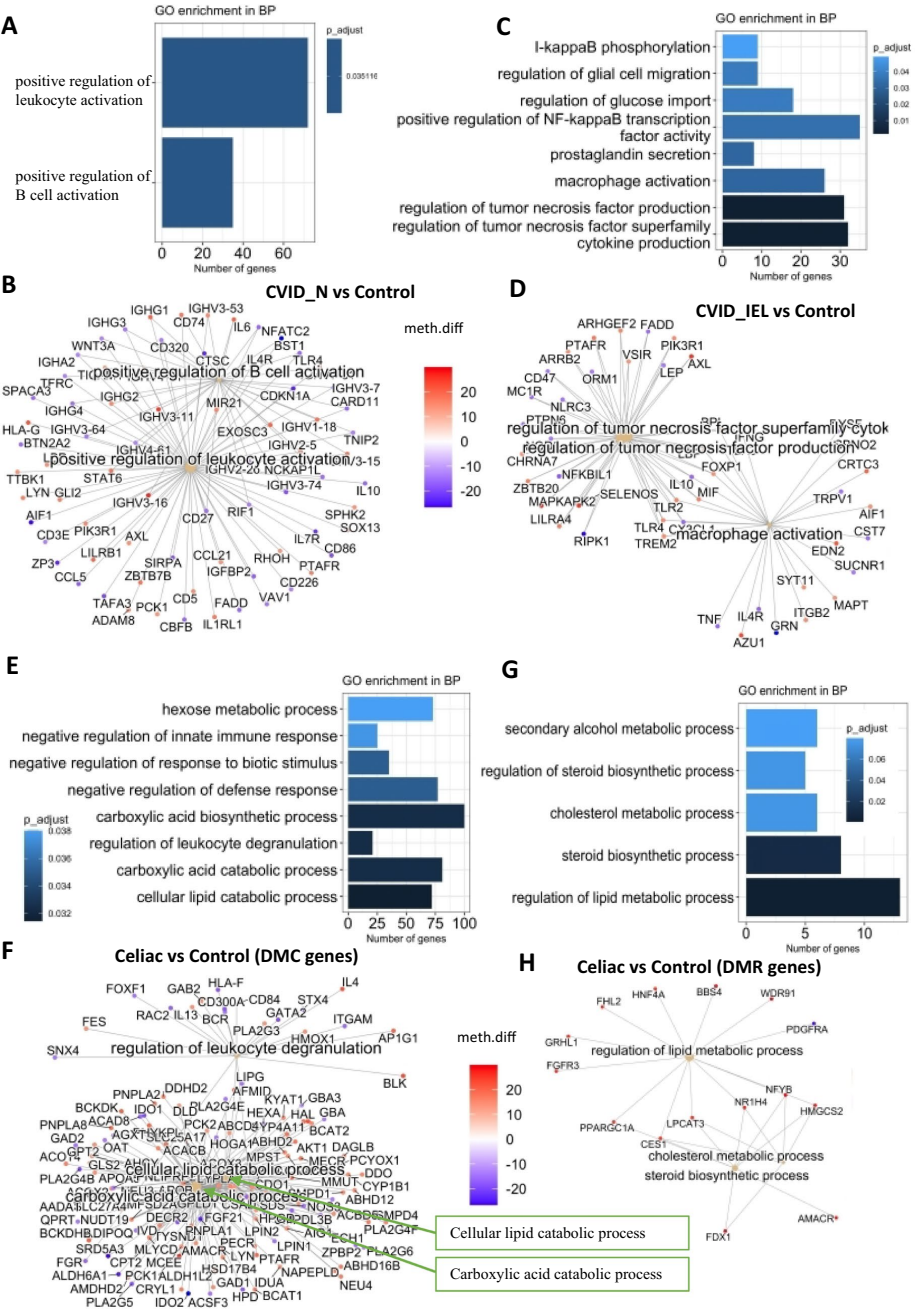
**Gene Ontology (GO) enrichment analysis for differential methylation associated genes. (A-F)** The top genes associated with differential methylation **(DMC)** for CVID_N, CVID_IEL and celiac disease compared to Controls **(G-H)** Genes associated with differential methylation in regions **(DMR)** in celiac disease vs Controls. No significant enrichment was identified in CVID_N_vs_Control or CVID_IEL_vs_Control for DMR. Bar plots showing GO enrichment terms in biological process (BP) (A, C, E & G) A net plot showing the protein interaction network in BP among identified DMC (B, D, F) and DMR (H) genes. The BP that was enriched were related to the regulation of B cell activation and leukocyte activation. BP, biological process; CVID_N, CVID patients with normal duodenal biopsies. CVID, Common variable immunodeficiency; Control: healthy controls with normal duodenal biopsies; Celiac, celiac disease, Marsh grade 3a-c, CVID_N, CVID patients with normal duodenal biopsies; CVID_IEL, CVID patients with duodenal inflammation

To assess the potential impact of DMCs on cellular function, we conducted a GO term enrichment analysis on the prominent DMC genes (defined as top DMC gene in this study). The GO analysis revealed that the top DMC genes (a total of 3129) were enriched in *biological processes of B cell/leukocyte activation* when we compared CVID_N to healthy controls (Fig. 6A-B). Comparing CVID_IEL to healthy controls (total 3 166 top DMC genes), genes were enriched in regulation of *tumor necrosis factor (TNF) and cytokine production* (Fig. 6C-D). Finally, when analyzing the 5280 DMC genes in celiac disease patients compared to controls, we observed enrichment in biology processes related to *lipid catabolism and regulation of leukocyte degradation* (Fig. 6E-F). GO enrichments analyses were carried out on the 139 DMR associated genes for celiac disease patients compared to controls. Our analysis consistently showed DMR associated genes enrichment in regulation of *lipid metabolic process, cholesterol metabolic process* and *steroid biosynthetic process* (Fig. 6G). For the two other patient groups, the number of DMR genes compared to controls were less than 50, which were too few to perform GO enrichment analysis.

### No clear differences in immune cell proportions between CVID, celiac disease and controls

To explore the cell subsets of tissue sample in the methylation sequencing data, we performed deconvolution analysis (in-silico analysis). The cell type was predicted by using a R package granulator (see methods). The result showed that the main detected immune cells included naive and memory CD4 + T cells, naïve CD8 + T cells, neutrophils and mucosal-associated invariant T cells (MAIT cells). A small proportion of naive and memory B cells was also detected. Importantly, the proportion profiles were very similar among control, CVID and celiac groups. Thus, no clear differences existed in cell proportion between the groups, suggesting that the methylation difference observed in this study does not merely reflect differences in immune cell distribution (Supplementary Figure S6 and Supplementary data 8).

### Comparing the methylation data with the previous published RNA-seq data

Finally, to provide a more comprehensive understanding of the molecular mechanisms we compared our previously published RNA-seq data [8] with the methylation data from this study. First, we identified the common genes that showed differential methylation (carried at least 2 DMCs) in our WGBS data and compared them to differentially expressed genes (DEGs) in our previous RNA-seq analysis. We identified five common genes from a total of 30 DEGs in CVID_IEL vs Control, and 15 common genes out of total 62 DEGs in Celiac vs Control, but no common genes were identified between CVID_N and controls (Supplementary data 9). Second, we performed correlation analysis between differential methylation and RNA expression using Spearman’s correlation analyses [23, 24]. The correlation was significant for Celiac vs Control (R = -0.78, p-value = 0.001). The correlation value, R, was also high for CVID IEL vs Control (R = -0.80), but the P value was not significant (P = 0.13), Supplementary Figure S7. The high correlation value combined with a non-significant P-value in the latter comparison is probably secondary to the low numbers of genes included in this analysis. Nonetheless, there appears to be a negative correlation pattern suggesting that lower methylation is associated with higher gene expression and vice versa for both comparisons (CVID_IEL vs Control and Celiac disease vs Control).

## Discussion

Despite extensive research in recent years, only 10–30% of the CVID cases have been associated to monogenic causes, suggesting alternative mechanisms such as epigenetic changes [10, 11]. Such data are, however, scarce and data from the GI tract are lacking. GI manifestations are frequent in CVID, and the GI tract could serve as a crucial intersection between gut microbiota, environmental factors, and immunity in these patients [2]. The present study is, to the best of our knowledge, the first to demonstrate alterations of epigenetic DNA methylation in gut biopsies in CVID. Our main and novel findings were: (I) Genome-wide analysis of CpG methylation showed an extensively different pattern in duodenal biopsies between the three patient groups (CVID_N, CVID_IEL and celiac diseases) and healthy controls; (II) We identified potential biomarkers of disease in gene promoters and in the region of high-frequency differentially methylated cytosines (in single base resolution) close to transcription start site in both CVID_IEL and celiac disease; (III) Limited overlap of associated genes for DMC and DMR indicates distinct disease mechanisms between CVID_IEL and Celiac disease; (IV) Prominent DMC genes were enriched in regulation of TNF and cytokine production in CVID_IEL compared to healthy controls, further supporting that CVID_IEL represents an inflammatory subgroup of CVID patients.

Methylation of the promoter region may affect transcription activation. The gene APOA1-AS in promoter was identically hypo-methylated in all three replicates of CVID_N compared to the healthy control group. Hypomethylation of 5mC in the promoter region often results in increased mRNA expression [25], as also observed in this study. Gene silencing becomes stronger as the amount of CpG dinucleotides at promoters increases [26]. However, in recent years it has become evident that regulation of gene expression through DNA methylation is a dynamic process which occurs in a context-dependent manner [25]. The promoter of gene MIR646HG was hypo-methylated in both Celiac and CVID_IEL, but not identified in CVID_N. Thus, although little overlap in the epigenetic signature between CVID_IEL and celiac disease, hypo-methylation of MIR646HG may reflect some common mechanisms caused by duodenal inflammation in both disease groups.

There were few common associated genes for DMC and DMR between CVID_IEL and CVID_N compared to healthy controls. This is in line with our recent observation that CVID patients with increased IEL displayed alterations in transcription of genes involved in response to viral infections compared to the other CVID patients [8]. Interestingly, these findings support the notion that CVID_IEL and CVID_N may represent distinct disease entities, as evidenced by the differences in GO enrichment analyses observed between these two groups. GO enrichment analyses for CVID_N compared to controls identified biological processes of B cell/leukocyte activation, which is in line with the etiology of CVID. B cell dysfunction and immunodeficiency in the gut also appears to affect methylation in associated genes. In CVID_IEL compared to control, enrichment analyses identified regulation of TNF production, cytokine production and macrophage activation. These are all known to be features of inflammation characterizing the immune dysregulation in this CVID sub-group. We have previously shown that CVID patients with autoimmune and inflammatory complications have increased TNF levels in peripheral blood both at the cellular and plasma levels [27, 28]. Notably, the pathways that were enriched in celiac disease compared to controls did not overlap with those that were enriched in CVID_IEL compared to controls, despite the similar histology observed in the duodenal biopsies. The findings that epigenetic modifications related to TNF activation and macrophage activation in this subgroup of CVID holds the potential to introduce novel strategies for prevention and therapy in subgroups of CVID.

In addition to methylation sites identified in promotor, a number of high-frequency DMC genes may also have the potential to be biomarkers for prevention and therapeutic targets. Of particular interest are the top four high-frequency DMC genes and the high-frequency DMC genes overlapping with DMR and/or DMP genes. For example, hypermethylation of SLC39A5 in celiac patients was identified in DMP, DMR as well as in high-frequency DMC genes (ten DMCs found in TSS ± 10 kb region). In CVID_ EL patients, three genes (SLC6A19, ACOT7 in hyper; MIR3142HG in hypo) were identified in the categories of both DMR and high-frequency DMC. Notably, SLC6A19 that was hypermethylated in CVID_IEL, encodes a system B(0) transmembrane protein that actively transports most neutral amino acids across the apical membrane of epithelial cells. This gene was highly expressed on the mRNA level in normal duodenum tissues (NCBI database: https://www.ncbi.nlm.nih.gov/gene/340024). A potential weakness of the study is the low number of DMR detected, although a high number of DMC are identified in this study. This may be explained by the feature of low-density DMC distribution in patients. The distribution pattern showed that most of the associated genes carried only one to two DMC across the genome, we used the default setting of window size at 1 kb for DMR scanning by methylKit tool, thus there are very few DMC sites in most scanning windows. Increasing the window size may be a way to increase the number of DMR, but it is at the expanse of the reduction of resolution. The number of samples included in epigenetic studies based on WGBS are often limited by the expensive cost per sample and the large size of dataset generated per sample which also limits the analysis of large cohorts and multiple comparisons between different groups. We acknowledge that the low number of patients in this study may be considered a limitation, however there are data to support that three or more biological samples in each group appear to be sufficient e.g. Ziller et al. who recommends a coverage of 5–15 folds per sample and three biological replicates [29]. Loyfer et al*.* recently published an extensive study in Nature, where they created a DNA methylation atlas for normal human cell types based on WGBS [30]. In this study, they analyzed 43 different tissues and most of the groups were n = 3. They found that samples from different subjects of the same type of tissue were more than 99.5% identical when n ≥ 3 [30]. Nonetheless, to fully establish the epigenetic profile in the GI tract from CVID patients, we need validation cohorts from other centers.

Celiac disease patients were younger than controls, reflecting the normal average age onset of celiac disease. Aging may influence methylation profiles in blood [31], however we do not know if or how aging effect methylation in duodenal tissue. There are to our knowledge no tools for utilizing the GWBS datasets to explore the effect on aging on duodenal samples, which may be a potential limitation [30].

Deconvolution analyses showed no clear differences in immune cell proportion between the groups, suggesting that the methylation difference observed in this study does not merely reflect differences in immune cell distribution. Of note, we searched for packages that could identify epithelial cells as well as immune cells, but we could not find current available and updated packages for bulk data for GI tissue. The lack of data on the proportion of epithelial cells is another potential limitation of the study. A potential strength of the study is the approach of computing high-frequency DMC genes. For example, in comparison of CVID_IEL vs control, a number of DMCs [14–17] in the top four DMC genes formed clear clusters inside just about 1–2 kb short regions. This approach of searching high-frequency DMCs genes has the advantages of high base resolution, flexible in region setting (e.g., TSS ± 10 kb, based on the DMC density distribution) and robustness. In addition, it is an easy way to visualize the differential methylation clusters/strings (Fig. 4B, Fig. 5 and Supplementary data 7).

This is the first study to show epigenetic changes in the duodenal biopsies of CVID patients occur. We have also detected potential biomarkers of disease in promotors and in high-frequency DMC close to TSS. Our findings may open up for novel targets for disease prevention and therapy. The study also points to the potential use of epigenetic profiling as biomarker to discriminate different classes of CVID patients even before full-blown clinical manifestations, potentially paving the way for personalized regimens for prevention and therapy in CVID. However, at this stage we cannot conclude if the actual changes in the methylation profile is a cause or a consequence of the clinical and inflammatory phenotype in the CVID_IEL subgroup, or as we believe, a combination thereof. We have previously suggested a pathogenic loop between an inflammatory phenotype that also include altered gut microbiota and altered methylation profile of DNA that again could contribute the phenotype CVID_IEL [2].

### Supplementary Information

Below is the link to the electronic supplementary material.

Supplementary file1 (DOCX 10858 KB)

Supplementary file2 (XLSX 4173 KB)

Supplementary file3 (XLSX 15 KB)

Supplementary file4 (XLSX 81 KB)

Supplementary file5 (XLSX 33 KB)

Supplementary file6 (XLSX 35 KB)

Supplementary file7 (XLSX 812 KB)

Supplementary file8 (XLSX 78 KB)

Supplementary file9 (XLSX 13 KB)

Supplementary file10 (XLSX 12 KB)

## Data Availability

The datasets analysed during the current study are not publicly available due to Norwegian legislation regarding general data protection regulation but are available from the corresponding author (SFJ), on reasonable request.

## Abbreviations

CpG methylation: The transfer of a methyl group to the cytosine, followed by guanine residues
CVID: Common variable immunodeficiency
CVID_IEL: Subclassification of duodenal biopsies from the CVID patient with increased intra epithelial lymphocytes
CVID_N: Subclassification of duodenal biopsies from the CVID patient with no increased intra epithelial lymphocytes (normal)
DMC: Differentially methylated cytosine
DMR: Differentially methylated region
DMP: Differentially methylated promoter
GI: Gastrointestinal
GO: Gene ontology
IEL: Intra epithelial lymphocytes
IFN: Interferon
TSS: Transcription start site
TNF: Tumor necrosis factor
WGBS: Whole genome bisulfite sequencing
5mC: 5-Methylcytosine
