# Supplementary material for: Altered Genome-Wide DNA Methylation in the Duodenum of Common Variable Immunodeficiency Patients
Source: J Clin Immunol. 2024 May 23;44(6):133. doi: 10.1007/s10875-024-01726-5 (PMC11116262; doi:10.1007/s10875-024-01726-5)
Supplement: Supplementary file 1 — Supplementary file1 (DOCX 10858 KB) [file 10875_2024_1726_MOESM1_ESM.docx]

**Altered genome-wide DNA methylation in the duodenum of common variable immunodeficiency patients**

Yang & Kaarbø *et al*

**Supplementary information**

Study design

Sample preparation for whole genome bisulfite sequencing (WGBS)

Whole genome bisulfite sequencing (WGBS) analyses

**Supplementary figures:**

Fig. S1 Workflow of WGBS data analysis

Fig. S2. Histograms showing the genome-wide read coverage distribution at CpG sites in the patients.

Fig. S3 Histograms showing the genome-wide methylation level profile at CpG sites in the patients.

Fig. S4 Bar plots showing the distribution of hyper- and hypomethylated regions in chromosomes.

Fig. S5. The distribution and frequency of differentially methylated cytosines (DMCs).

Fig. S6. Immune cell proportion predicted in biopsy by deconvolution in bulk RNA-seq.

Fig. S7. The correlation between differential methylation and gene expression.

**Supplementary data list:**

Data 1. Identified DMC associated genes.xlsx

Data 2. Identified DMP genes.xlsx

Data 3. Identified DMR associated genes.xlsx

Data 4. Common DMC and DMR genes in CVID and celiac patients.xlsx

Data 5. Unique DMR genes.xlsx

Data 6. Top DMC associated genes.xlsx

Data 7. High frequency DMC genes.xlsx

Data 8. Cell types deconvolution in RNA-seq.xlsx

Data 9. Common genes in DMCs and RNA-seq DEGs.xlsx

**Supplementary figures:**

**Supplementary information**

**Study design**

The study included an upper endoscopy (GIFHQ190, Olympus, Hamburg, Germany). Intestinal biopsies were collected according to protocol^1^ at the Section for Gastroenterological Endoscopy at Oslo University Hospital (OUH), Rikshospitalet, Oslo, Norway. The biopsies from the CVID patients were collected, and classified, as previously described^1^. An increased number of IELs in the duodenal mucosa was defined by a number of IEL ≥25 per 100 enterocytes^2,3^. The patients with celiac disease had Marsh grade 3a-c, and the biopsies were taken before treatment (gluten free diet). Healthy controls were recruited from individuals that were referred to the same endoscopy unit. Prior to inclusion as a healthy control, referrals were assessed by KEAL and SFJ for eligible candidates. Individuals with a low suspicion of GI disease, and with no other medical history, were invited by letter to participate in the study a few weeks before their planned endoscopy. The reasons for referral were typically abdominal pain or suspicion of gastroesophageal reflux. At the day of the endoscopy a more thorough medical history was taken and only individuals that were otherwise healthy and on no medication were included. Afterwards, we excluded individuals if routine blood screening (including liver function tests, creatinine, Hb, leukocyte count, C-reactive protein and immunoglobulin levels) taken on admission was abnormal. Also, duodenal biopsies that were not described as normal by the pathologist were excluded. Finally, from the included healthy controls we selected three to best match the sex and age distribution of the included CVID patients.

We compared duodenal biopsies from three patient groups: (i) CVID patients with IEL, CVID_IEL; (ii) CVID patients *without* IEL, CVID_N; (iii) patients with untreated celiac disease (similar duodenal inflammation as in CVID patients) with healthy controls. By including a group of CVID patients without duodenal inflammation, we can also explore the difference in CVID unrelated to the presence of inflammation, and if there are similar changes in CVID and celiac disease related to inflammation which is not disease specific.

**Sample preparation for whole genome bisulfite sequencing (WGBS)**

DNA was isolated from snap frozen biopsies of duodenal biopsies (n=3-5) using DNA, RNA and protein Allprep kit (Qiagen, Hilden, Germany) according to the manufacturer’s instructions with a few modifications. The samples were lysed in RLT buffer with β -mercaptoethanol and homogenized using a QIAshredder (Qiagen). Genomic DNA was eluted in molecular grade H_2_0 and stored at -20°C. Genomic DNA was quantified with Qubit 2.0 DNA HS Assay (ThermoFisher, Massachusetts, USA) and quality assessed by Tapestation genomic DNA Assay (Agilent Technologies, California, USA).

**Whole genome bisulfite sequencing (WGBS) analyses**

WGBS was performed by Admera Health, NJ, USA. Genomic DNA was bisulfite converted with the EZ DNA Methylation-Gold Kit (Zymo Research, California, USA) following manufacturer’s protocol. Library preparation was then performed using Accel-NGS®Methyl-Seq DNA Library kit (Swift Biosciences, Michigan, USA) per manufacturer’s recommendations. Final libraries quantity was assessed by APA SYBR® FAST qPCR with QuantStudio ® 5 System (Applied Biosystems, California, USA) and quality was assessed by TapeStation HSD1000 ScreenTape (Agilent Technologies Inc., California, USA). Equimolar pooling of libraries was performed based on QC values. Samples were sequenced on an Illumina® HiSeq (Illumina, California, USA) with a read length of 150 bp and 300 M (millions) paired end (PE) reads per sample.

The initial processing data of genome-wide 5mC counts was generated by Admera Health. Briefly, the following steps were included: the raw data of FASTQ files was cleaned by Trim Galore (version 0.6.5) and subjected to quality control by FastQC (version 0.11.9). The cleaned WGBS data (about 300 M PE reads per samples) was aligned to the Hg38 human reference genome, followed by extraction of genome-wide 5mC in CG, CHG and CHH context separately using Bowtie2 (version 2.4.1)^4^and Bismark (version 0.22.3)^5^. Next, we used the methylKit R package (version 1.16.1)^6^ to analyze the generated BAM alignment files and quantify levels of methylated bases of 5mC and identify differently methylated cytosines (DMCs), regions (DMRs), and promoters (DMPs) in CG context. The parameter settings for methylKit were: the minimum read coverage at each CpG site ('mincov') is 10 reads at each DMC; the minimum number of covered bases per region ('cov.bases') is 5 and 10 in each DMR and DMP, respectively; the tiled window ('win.size') is 1000 bp for DMR. The DMP regions were defined as the transcription start site (TSS) +/- 1000 bp. The genome annotation database used in the DMP analysis was the biomaRt R package (version 2.54.0)^7^. Significantly different methylation between groups (i.e., hyper or hypo) was defined by a mean methylation difference ≥ 25% and an adjusted q-value ≤ 0.01. The detail of statistic approach for differential methylation analysis is described in the section of “Statistical analysis**”.** The top DMC (associated) genes were defined by the subset of DMCs with mean methylation difference ≥ 30% and distance to TSS ≤ 10 kb. The high-frequency DMC (associated) genes were defined as the subset of DMC genes presenting a minimum of five DMCs per transcript in at least one transcript/isoform.

To avoid gender bias, the reads mapping to the X and Y chromosomes were removed in downstream analysis. The differential methylation analysis was performed on the Saga computer cluster provided by UNINETT Sigma2. Finally, Gene Ontology (GO) enrichment analysis of differentially methylated genes was performed by the clusterProfiler R package (version 4.6.0)^8^ using the over-representation function 'enrichGO' with a Bonferroni-Holm (BH) adjusted p-value threshold at 0.05. The GO terms in the categories of biological function (BF), molecular process (MP) and cellular component (CC) were analyzed separately. The genome annotation database used for GO analysis was the org.Hs.eg.db (R package, version 3.16.0). The data visualization was carried out using various R packages including tidyverse (version 0.4.2), VennDiagram (version 1.7.3), and ggplot2 (version 3.4.0) in Rstudio (http://www.rstudio.com/). The methylation profile in promoter regions were plotted by ggplot2 using the default smoothing method of auto detection. The bioinformatic analysis pipeline is summarized in (Supplementary Figure S1).

**Supplementary Figures:**


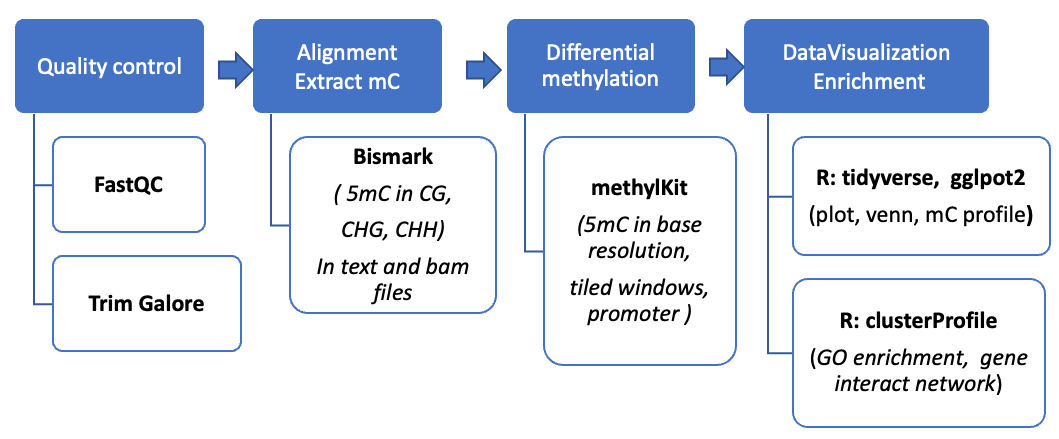


**Fig. S1 Workflow of WGBS data analysis.** Quality control of the raw reads was performed with FastQC. The adaptors and low-quality reads were removed with Trim Galore. The paired reads were then aligned against the reference genome, the counts of 5mC and unmethylated C in each position were extracted in the context of CG, CHG and CHH separately using the Bismark package. The methylKit tool was used for computing differential methylation at base resolution (DMC) and in regions (DMR) using tiled windows. The data was visualized using several R packages. The methylation curve was generated by ggplot2 using the 'geom_smooth' function. The Gene Ontology (GO) enrichment analysis was performed using the clusterProfiler R package. The software packages are shown in bold.

**CVID_N**

**CVID_IEL**

**Celiac**

**Control**


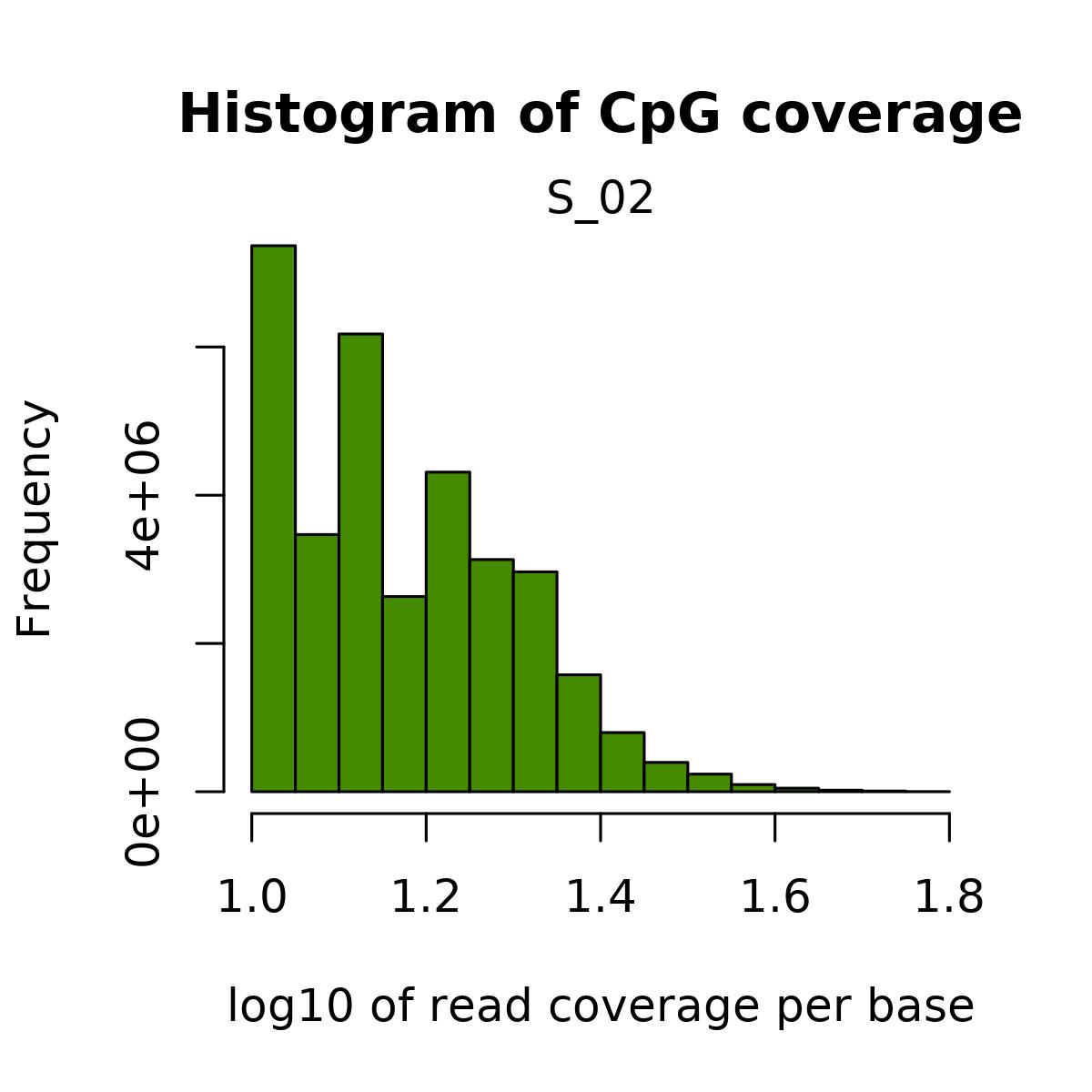

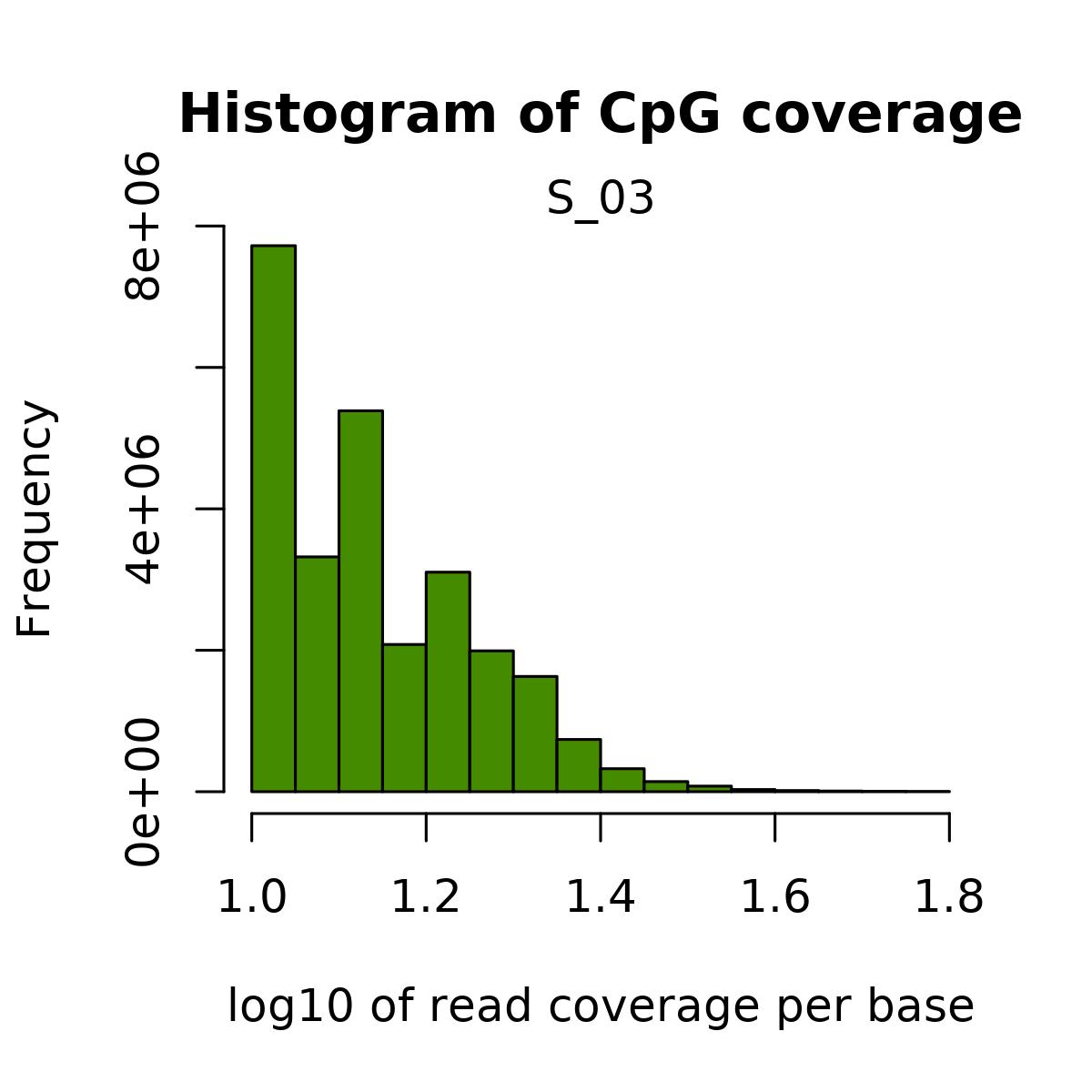

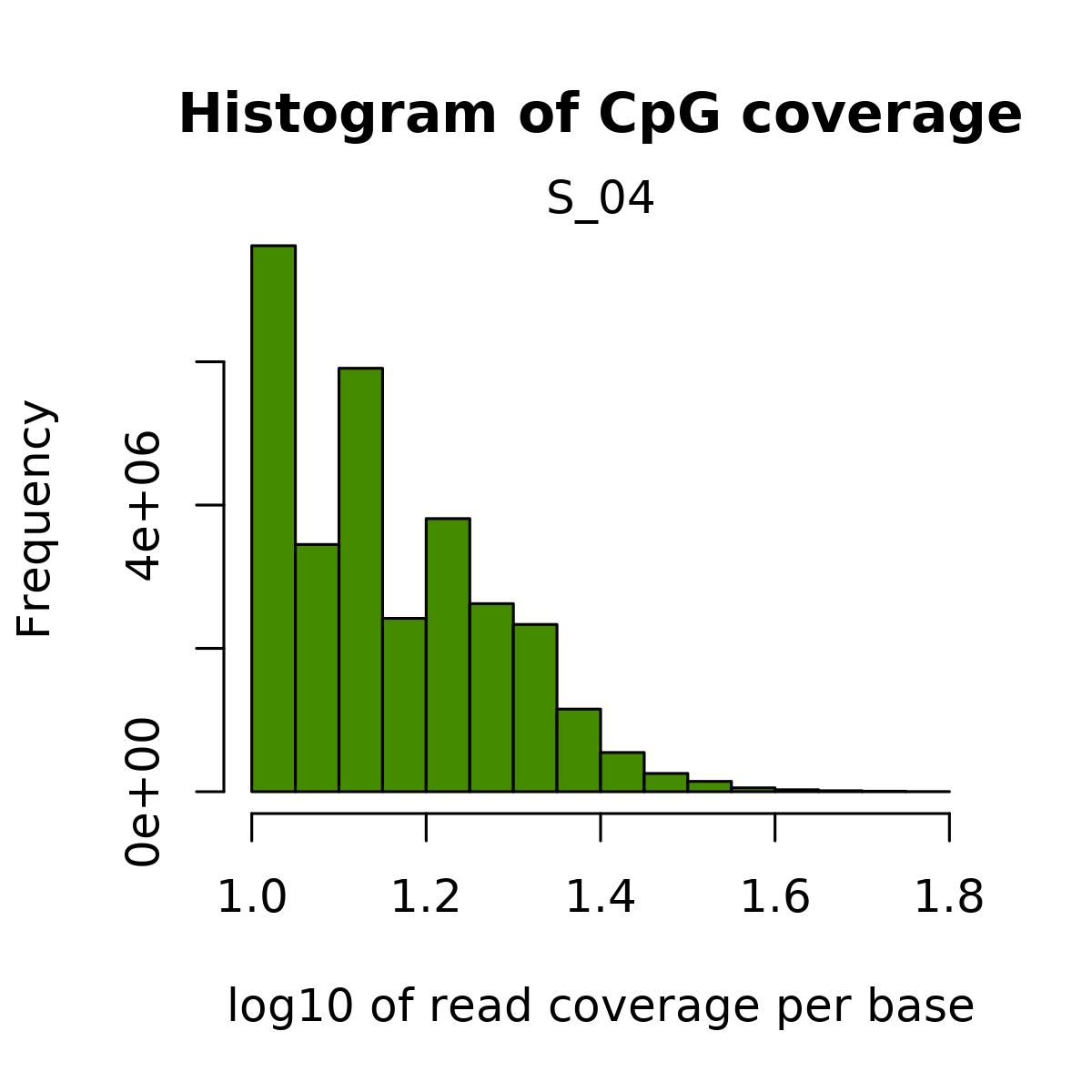

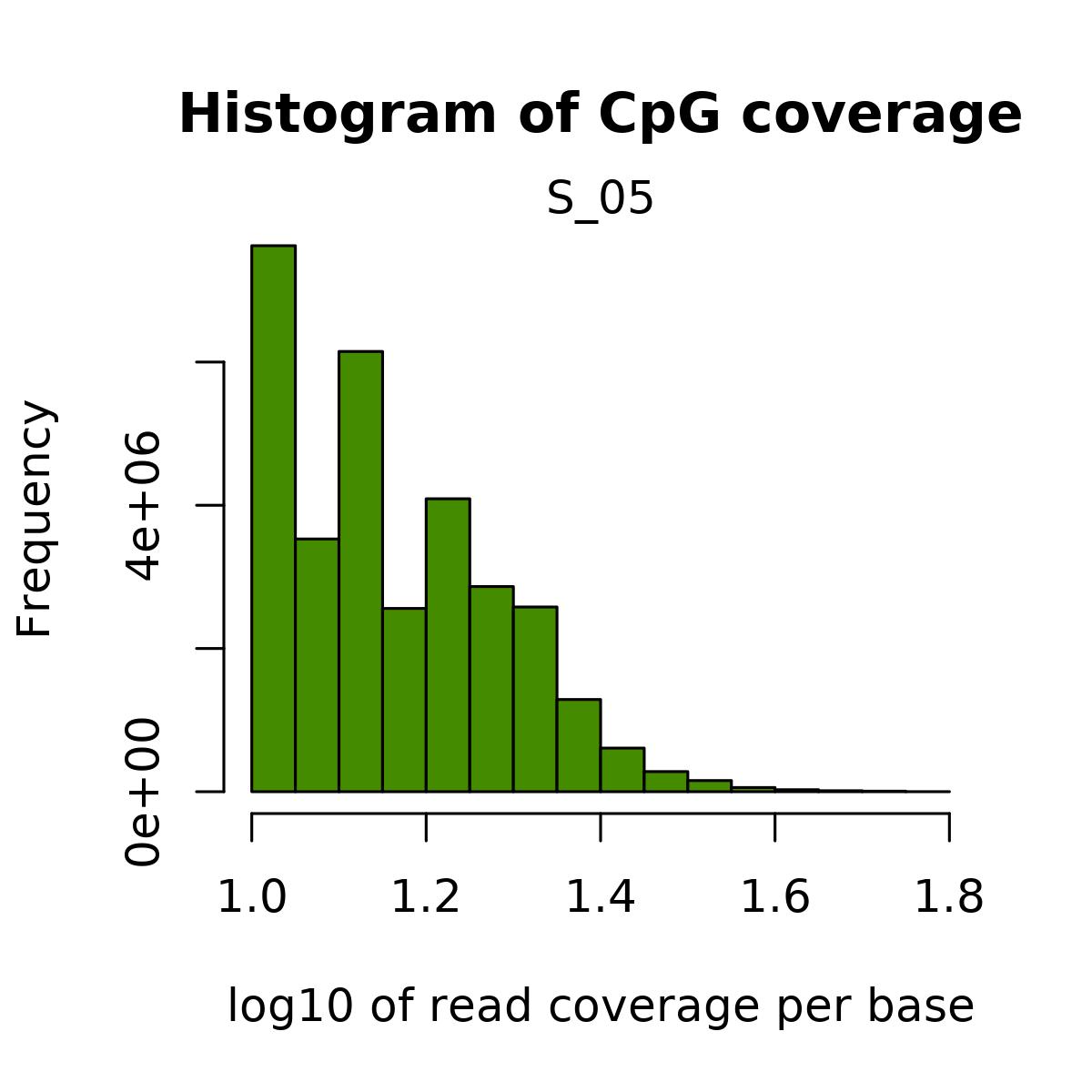

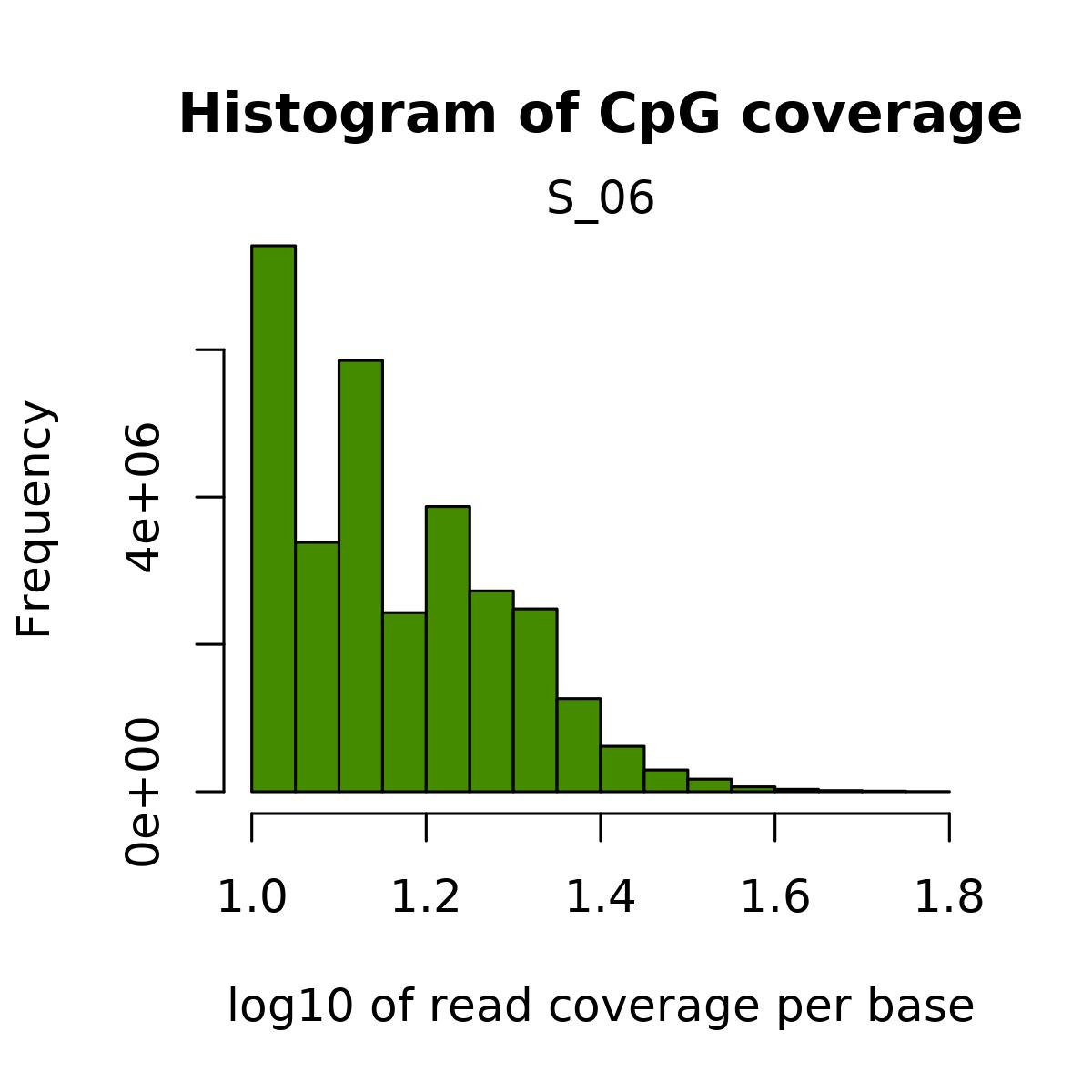

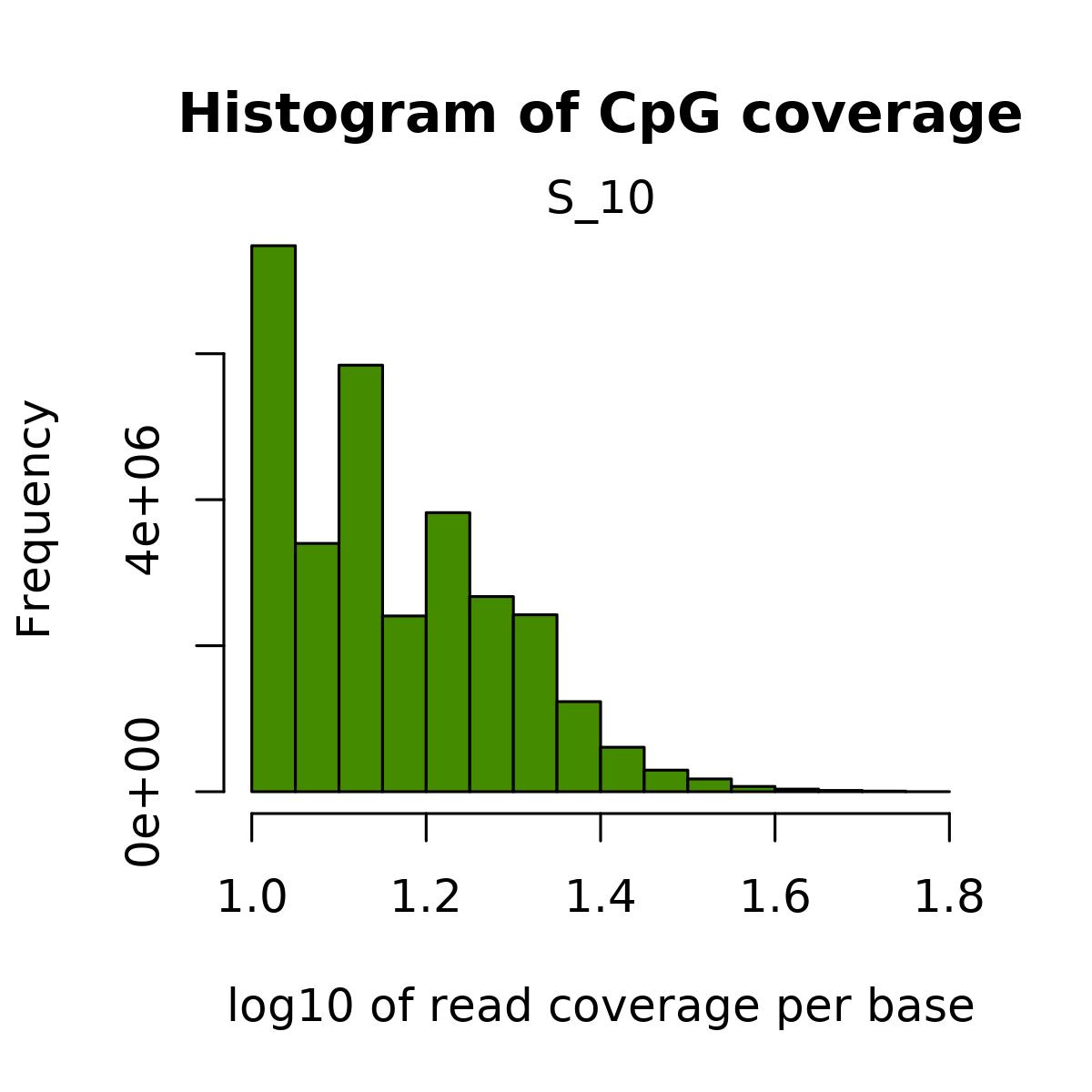

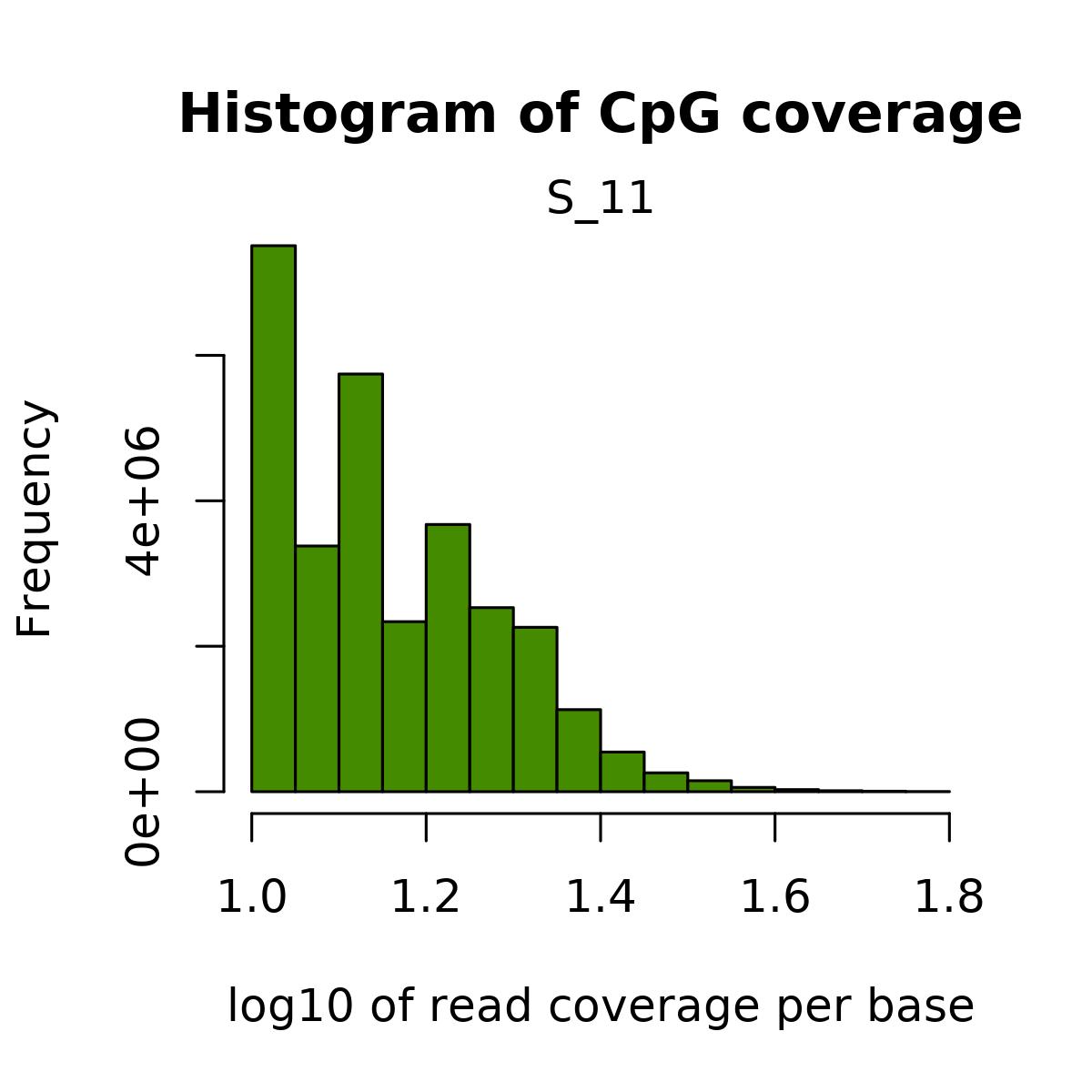

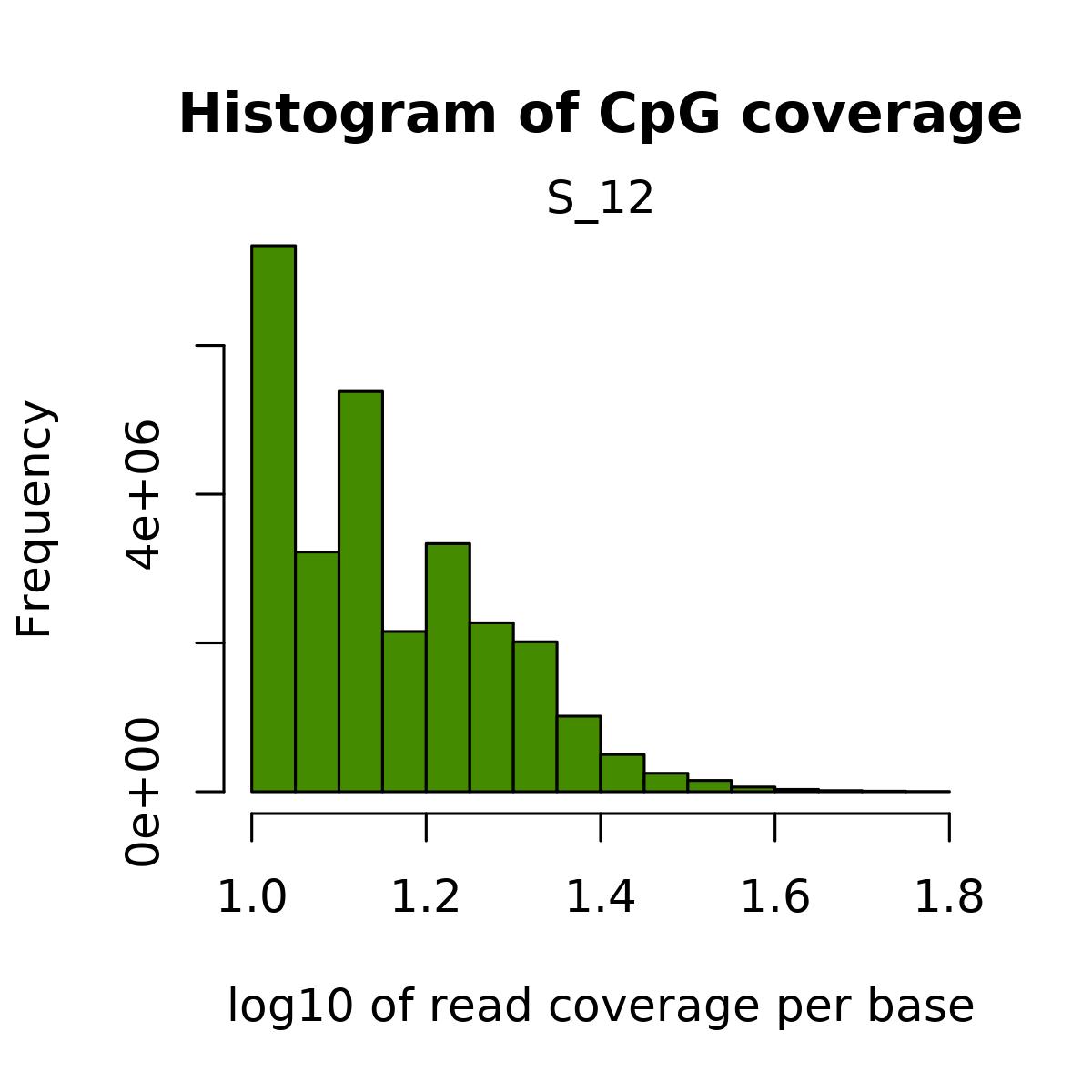

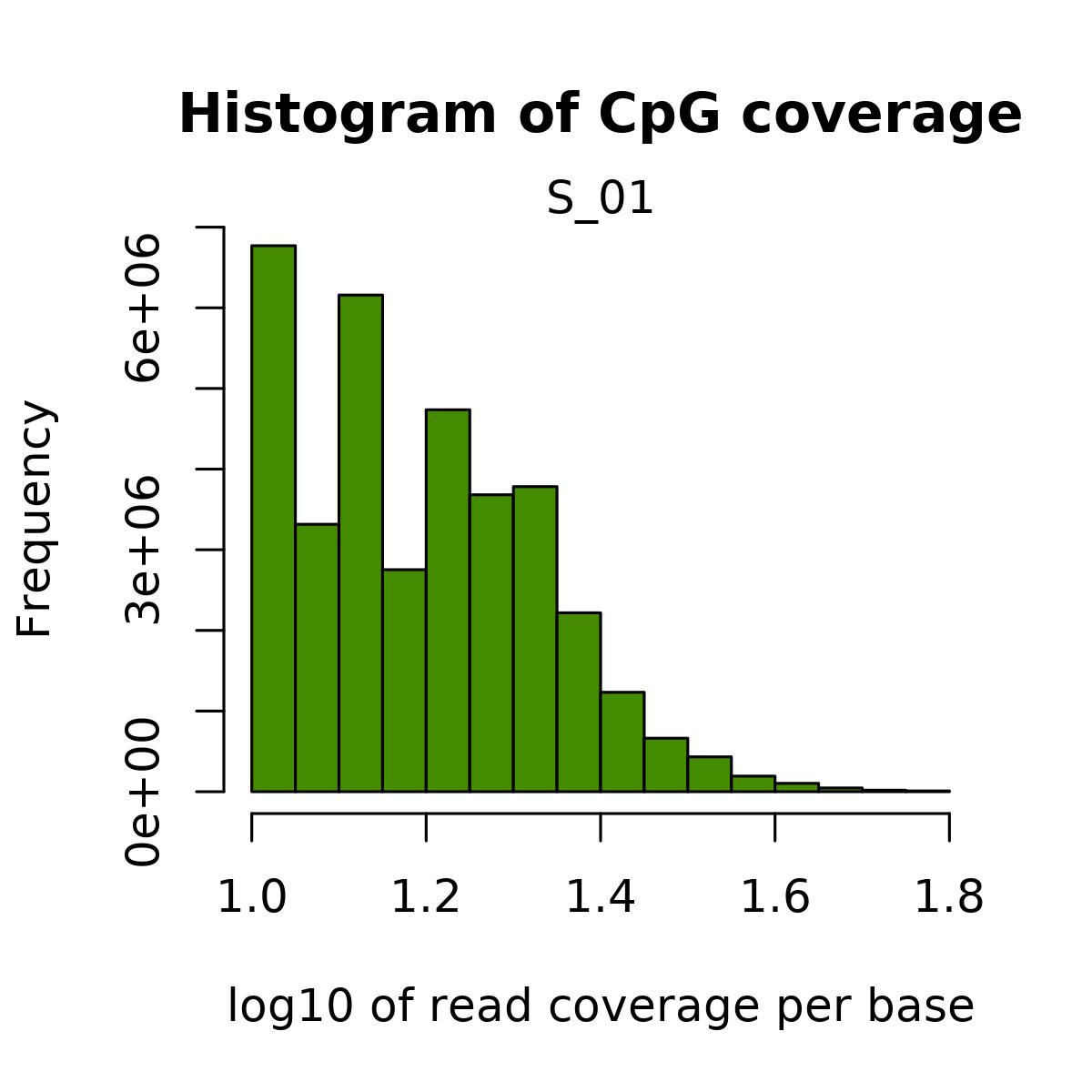

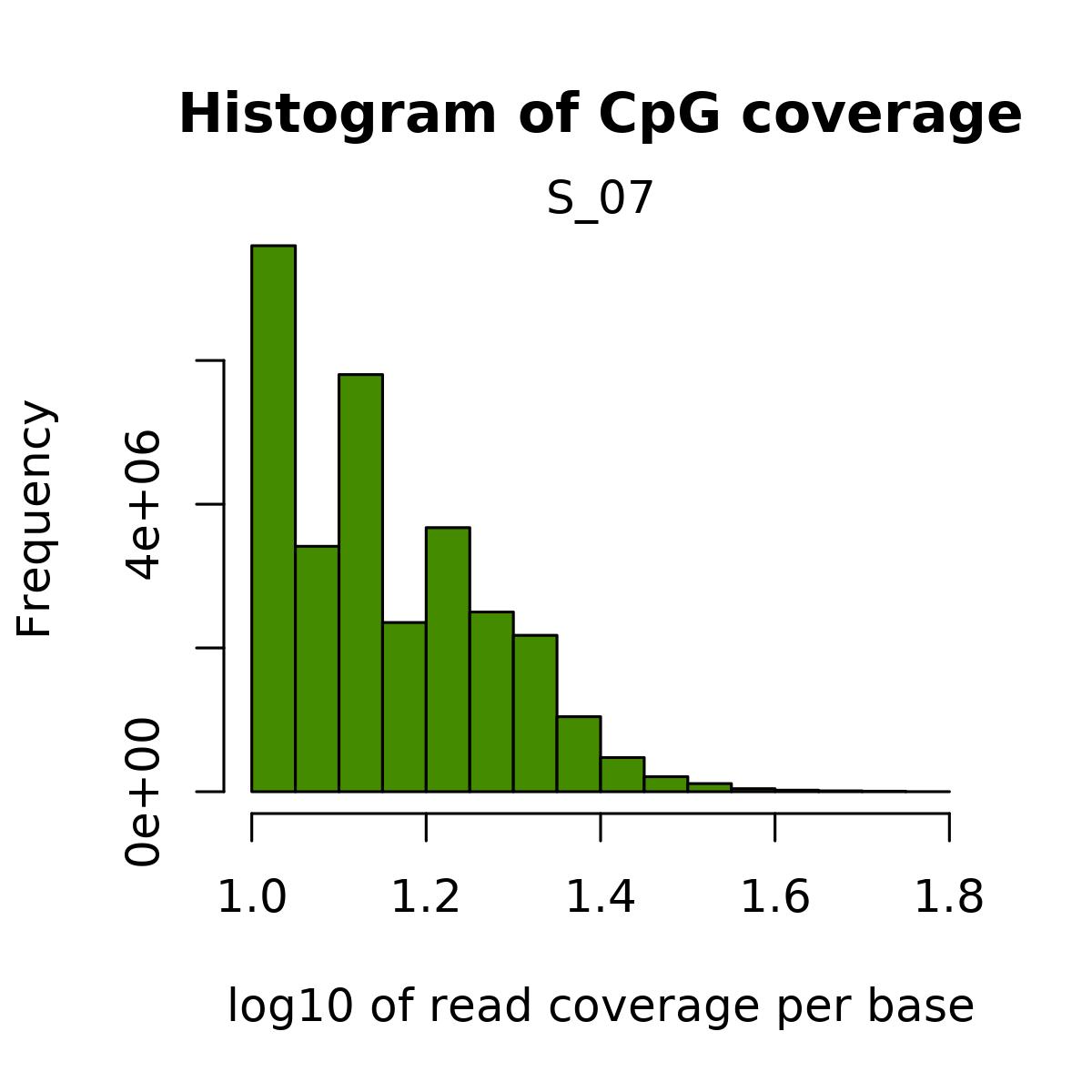

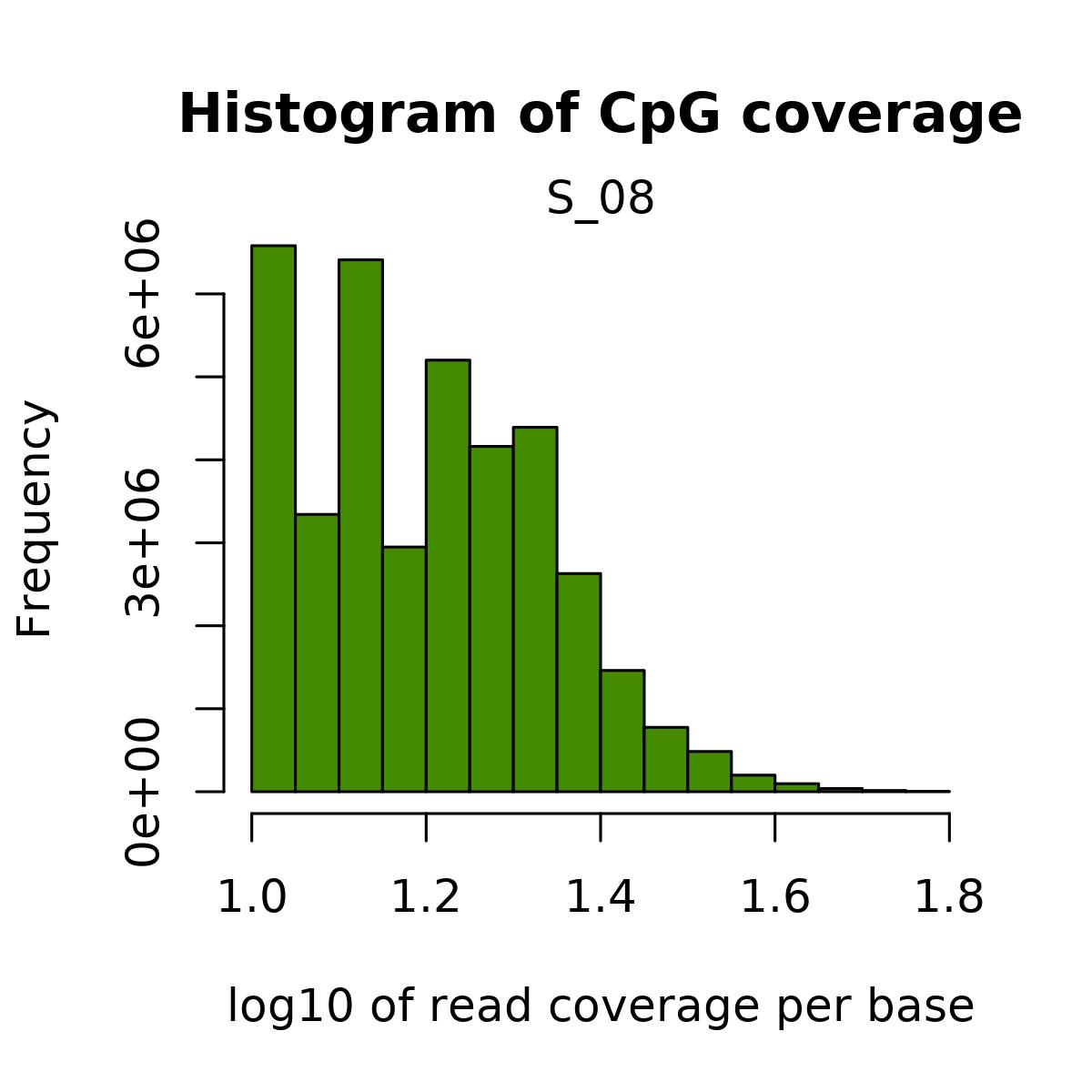

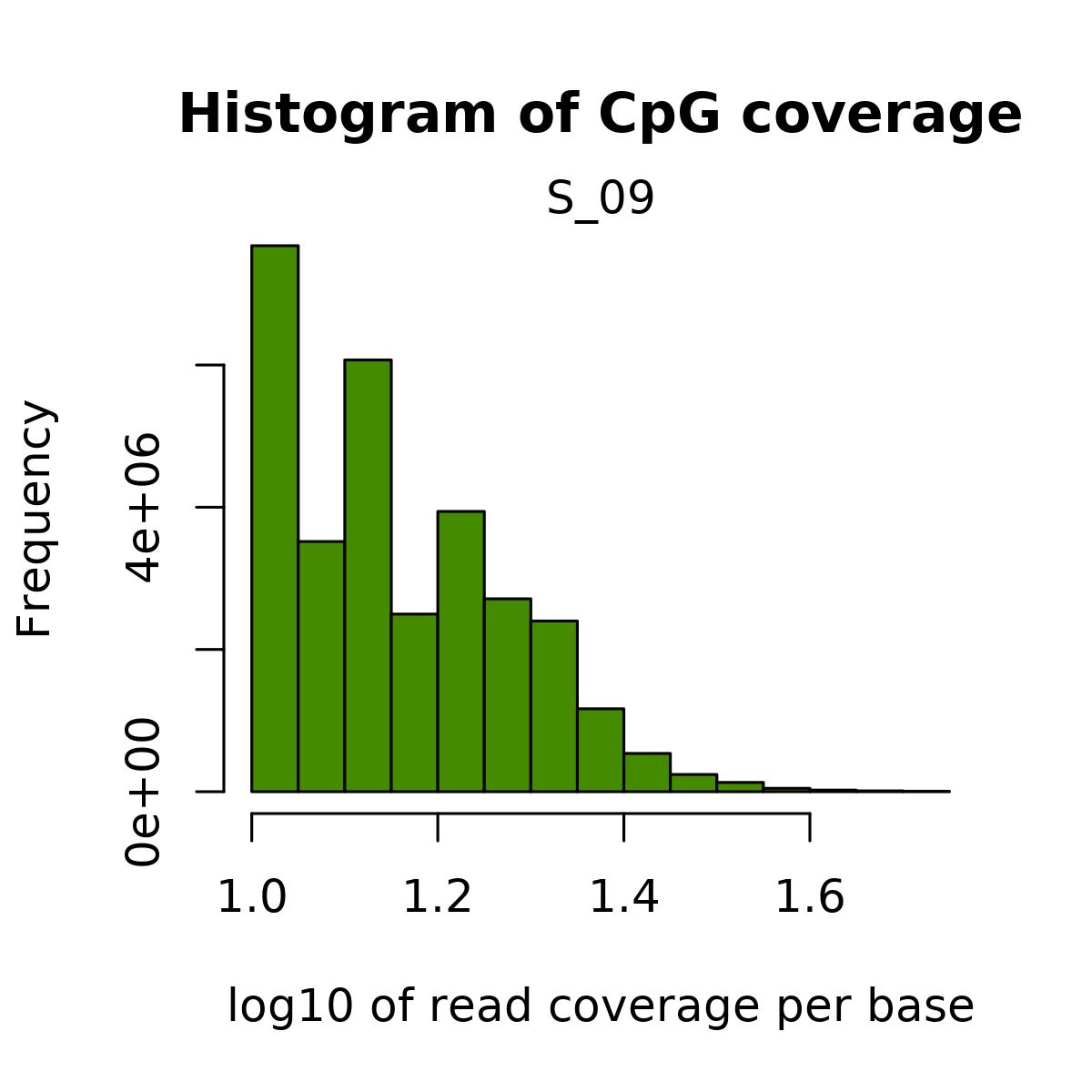

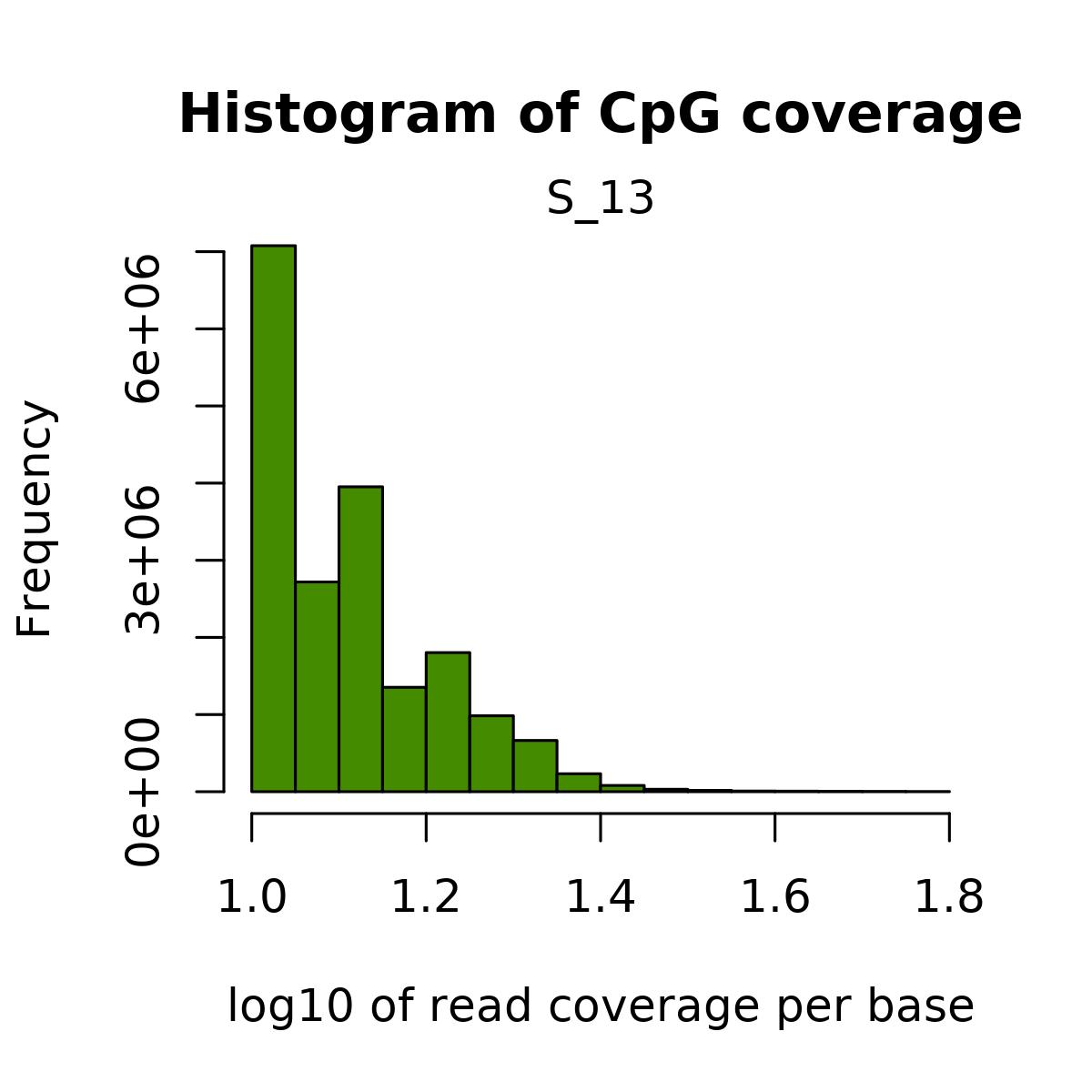

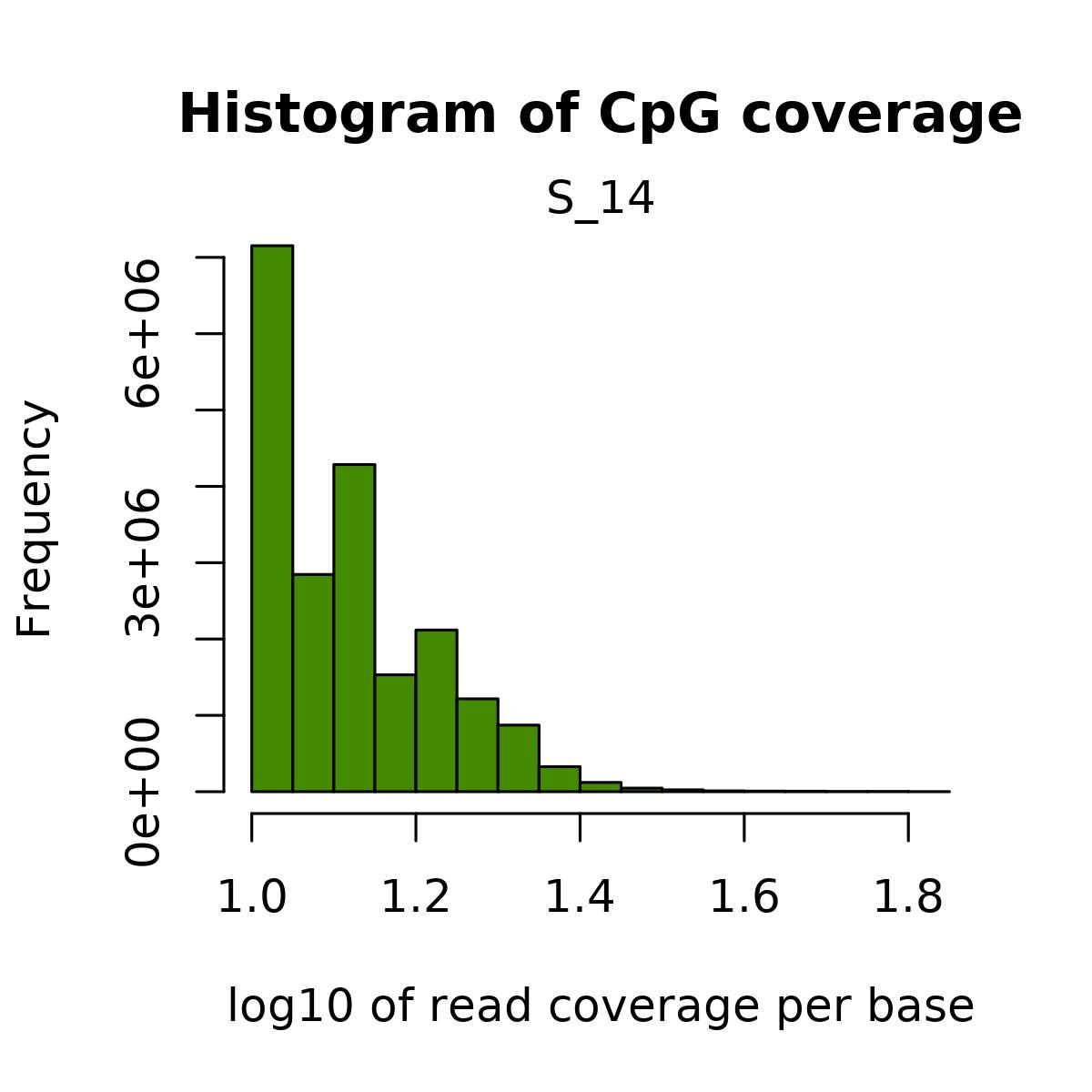


**Fig. S2.** **Histograms showing the genome-wide read coverage distribution at CpG sites in the patients.** The majority of sites have depth of 1-1.4 in log10 scale per site, corresponding to a depth of 10-25 reads per site. ­­

CVID: Common variable immunodeficiency; Control: healthy controls with normal duodenal biopsies; Celiac: celiac disease Marsh grade 3a, CVID_N: CVID patients with normal duodenal biopsies; CVID_IEL: CVID patients with duodenal inflammation.


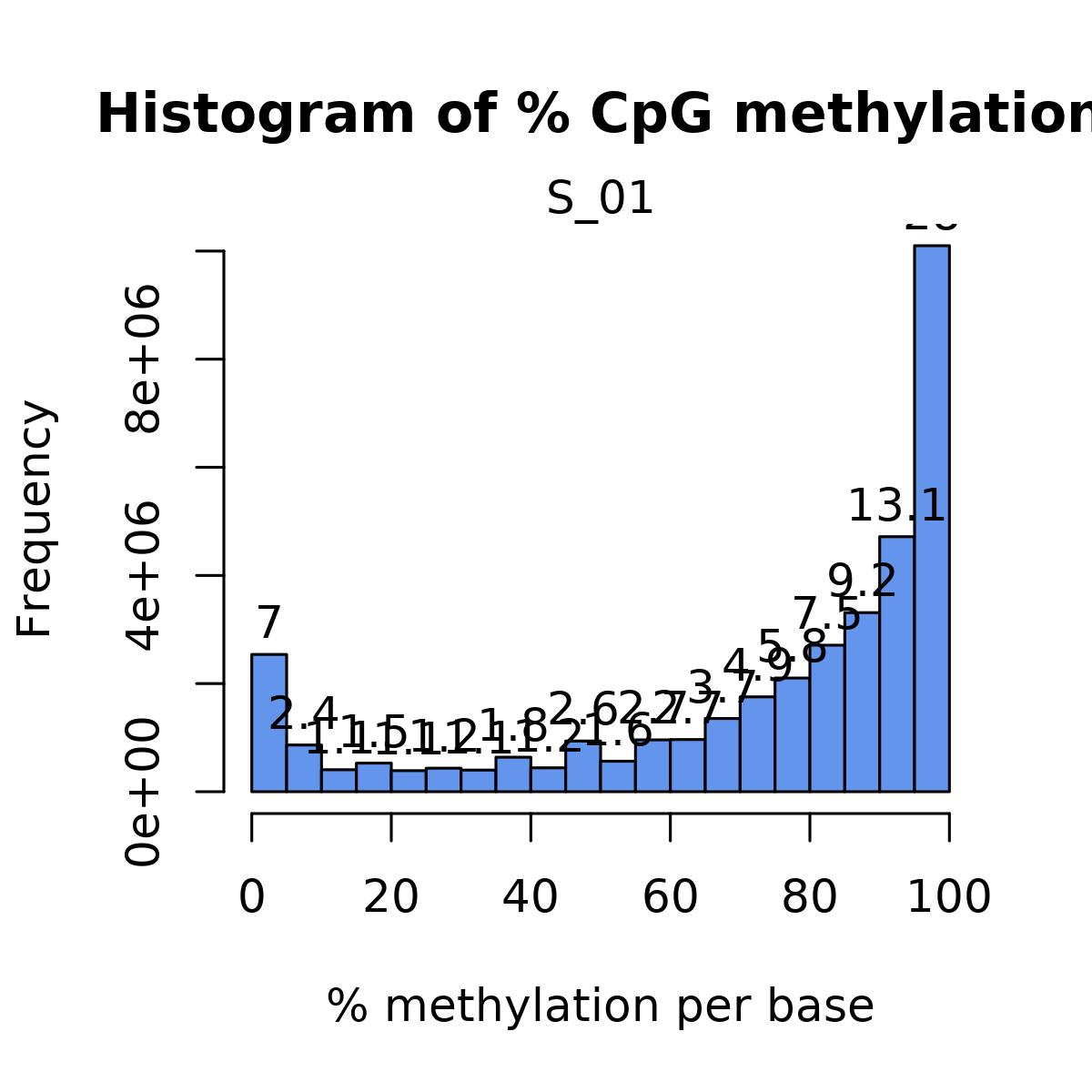

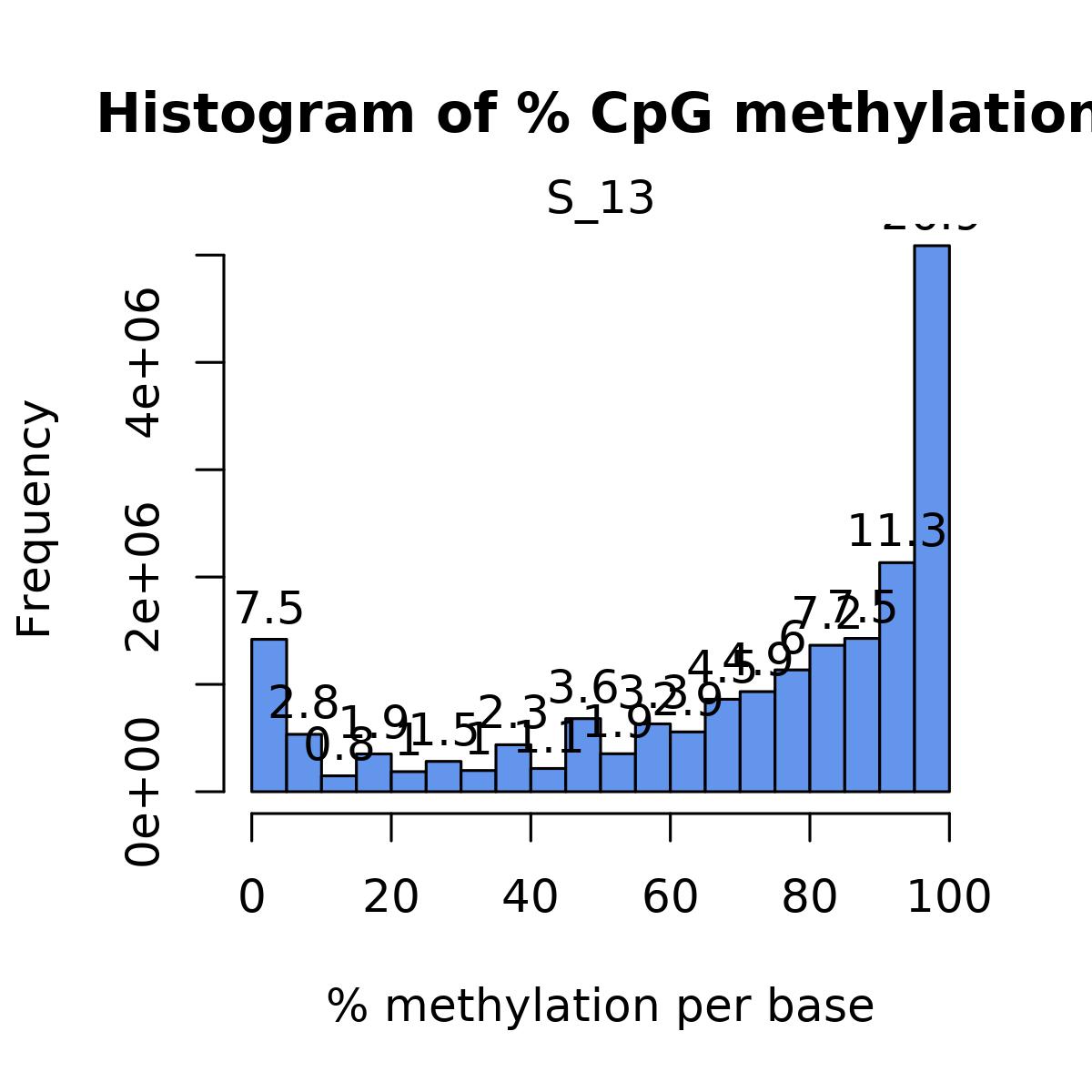

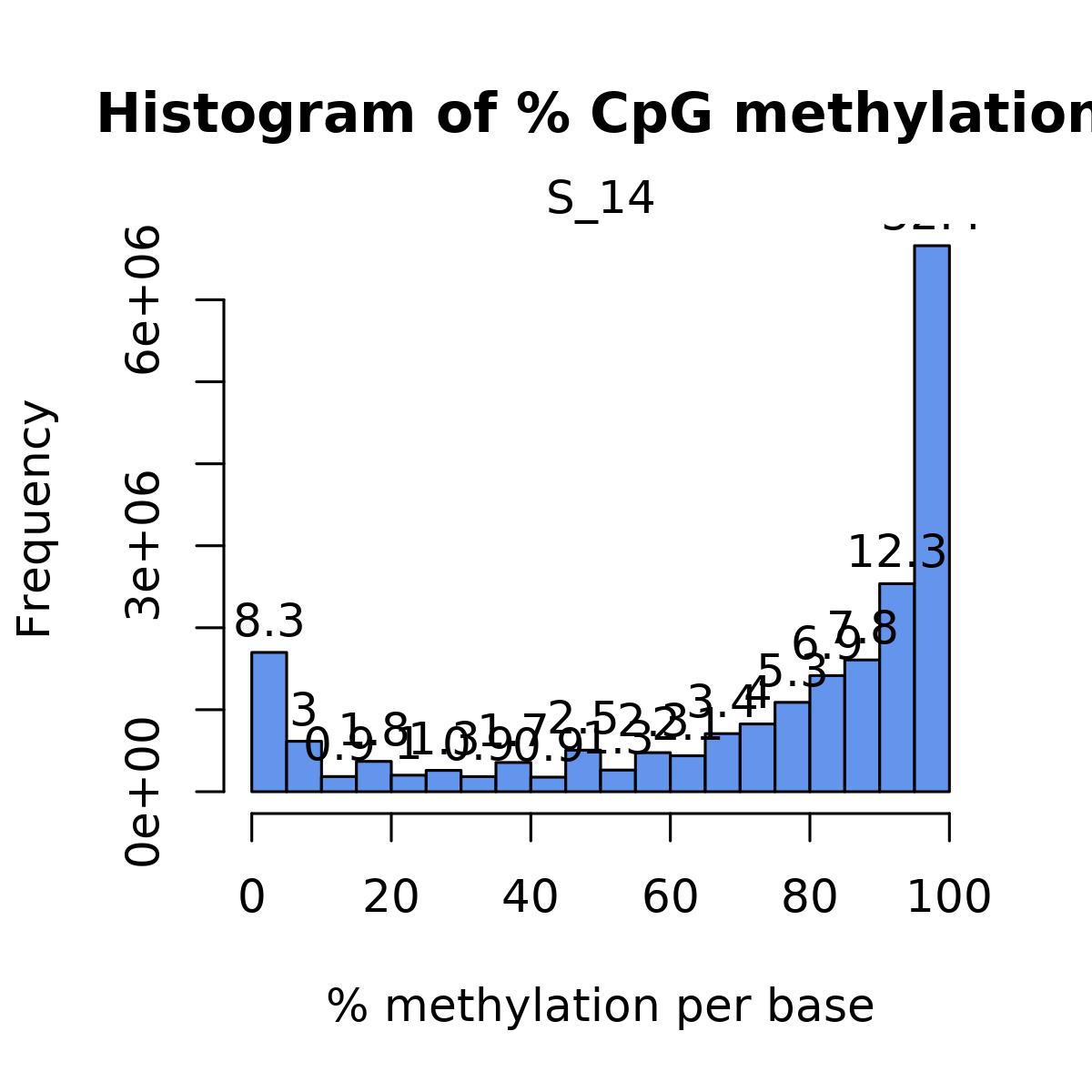

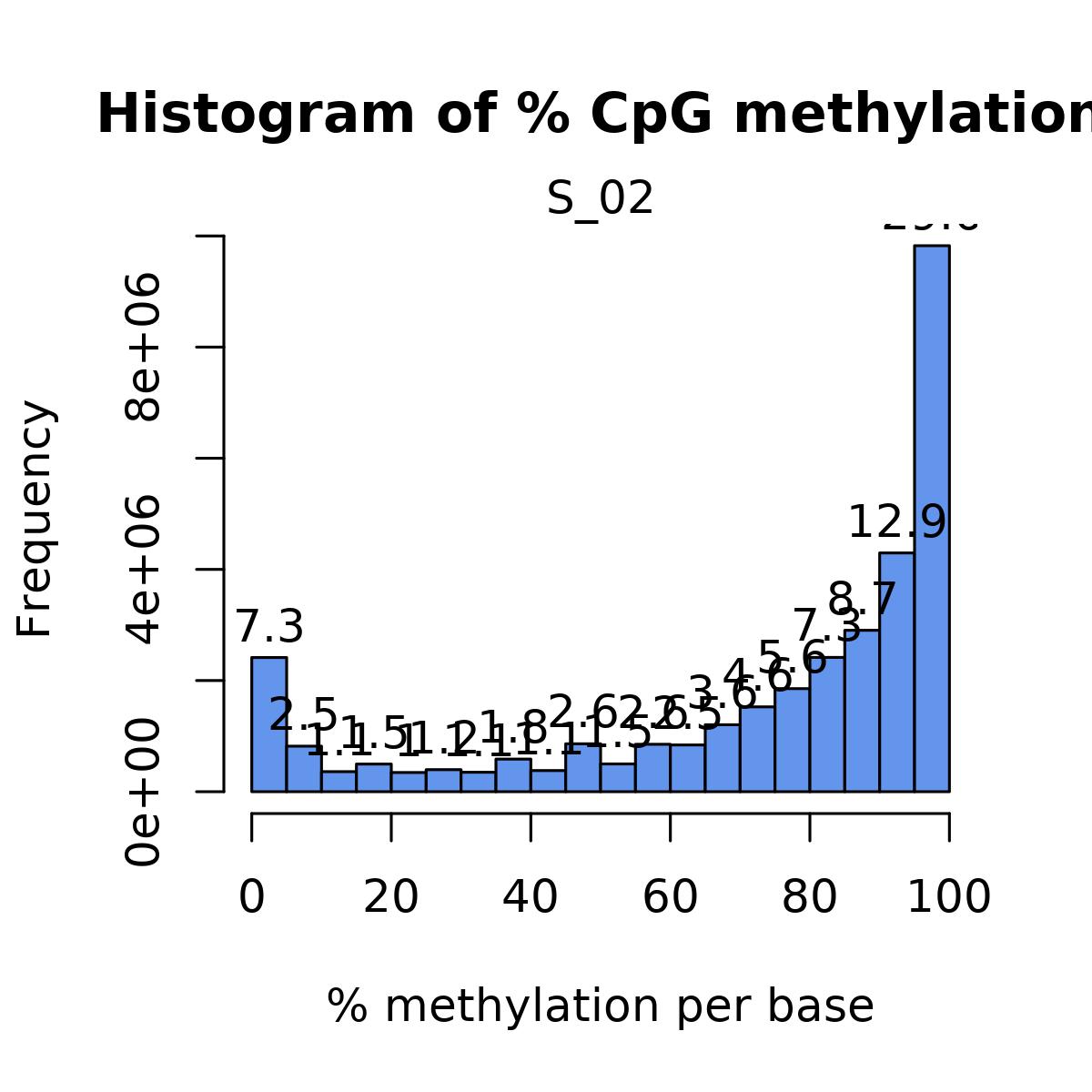

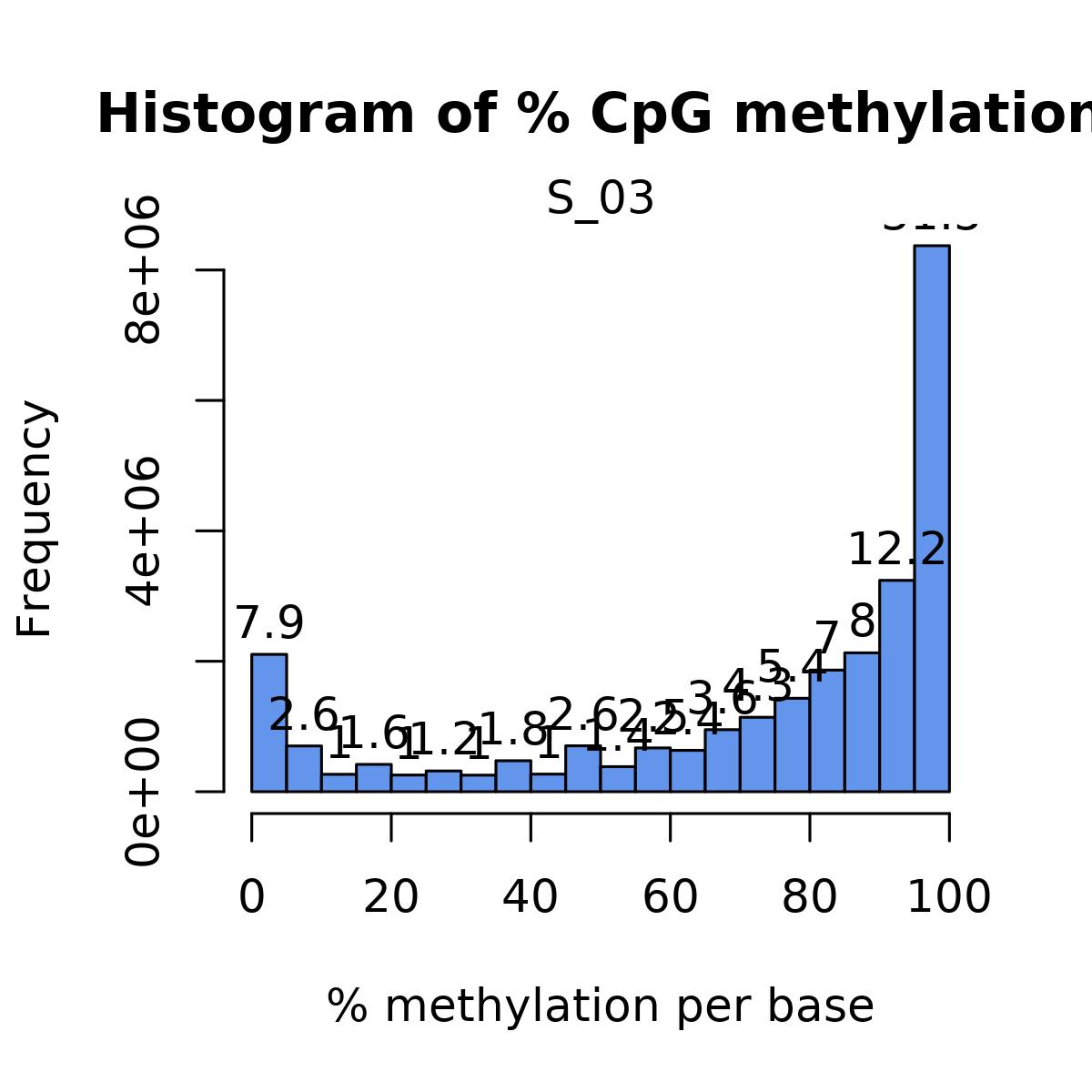

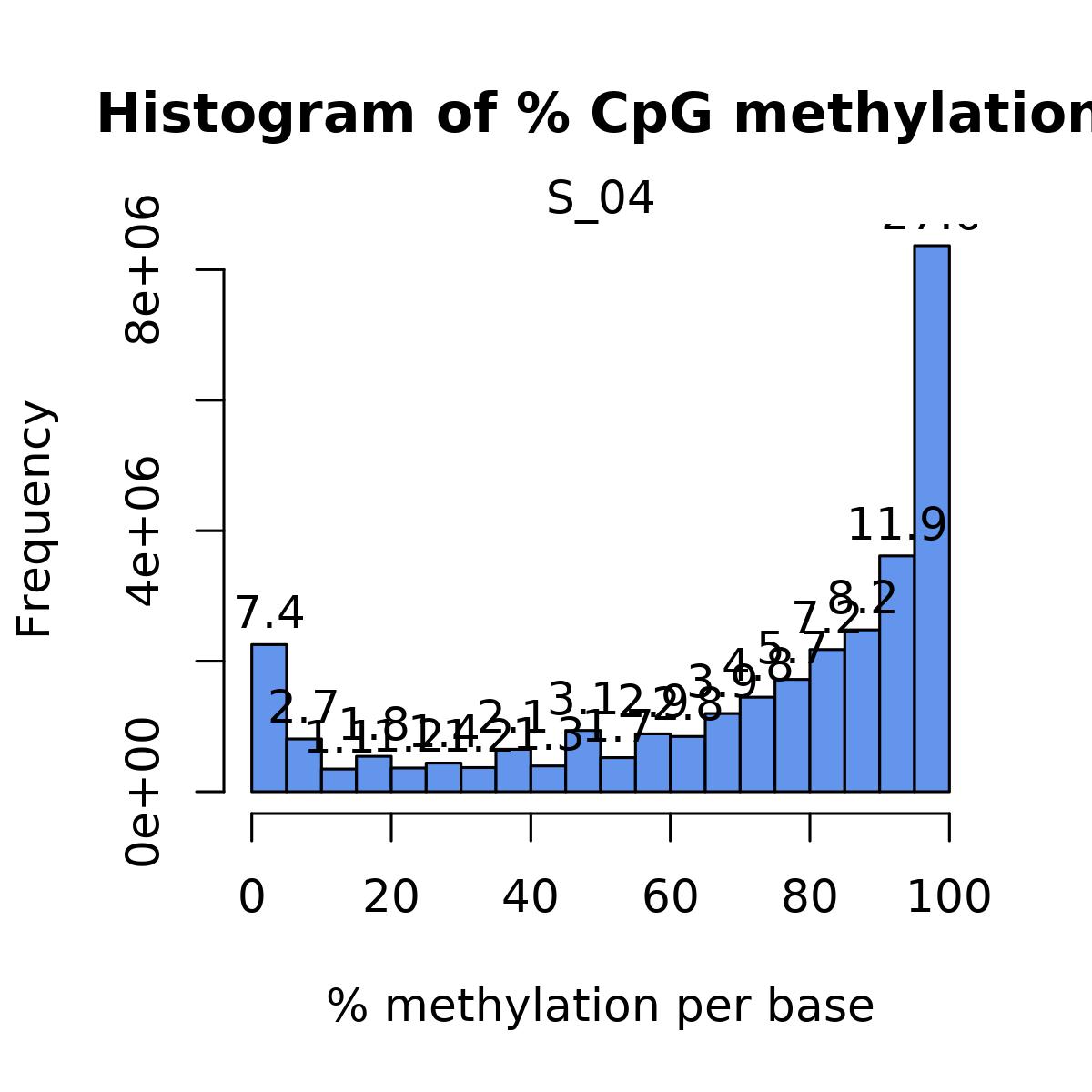

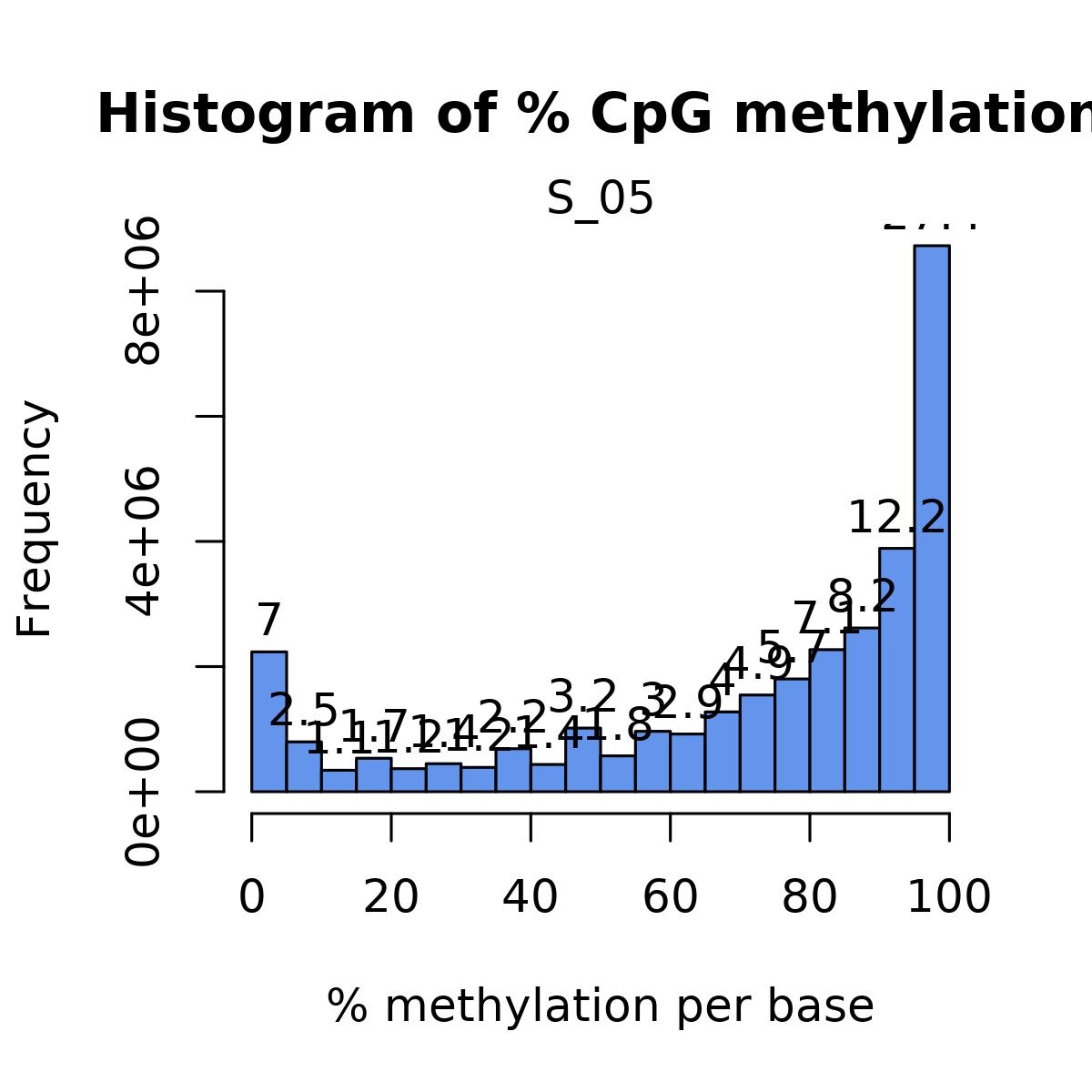

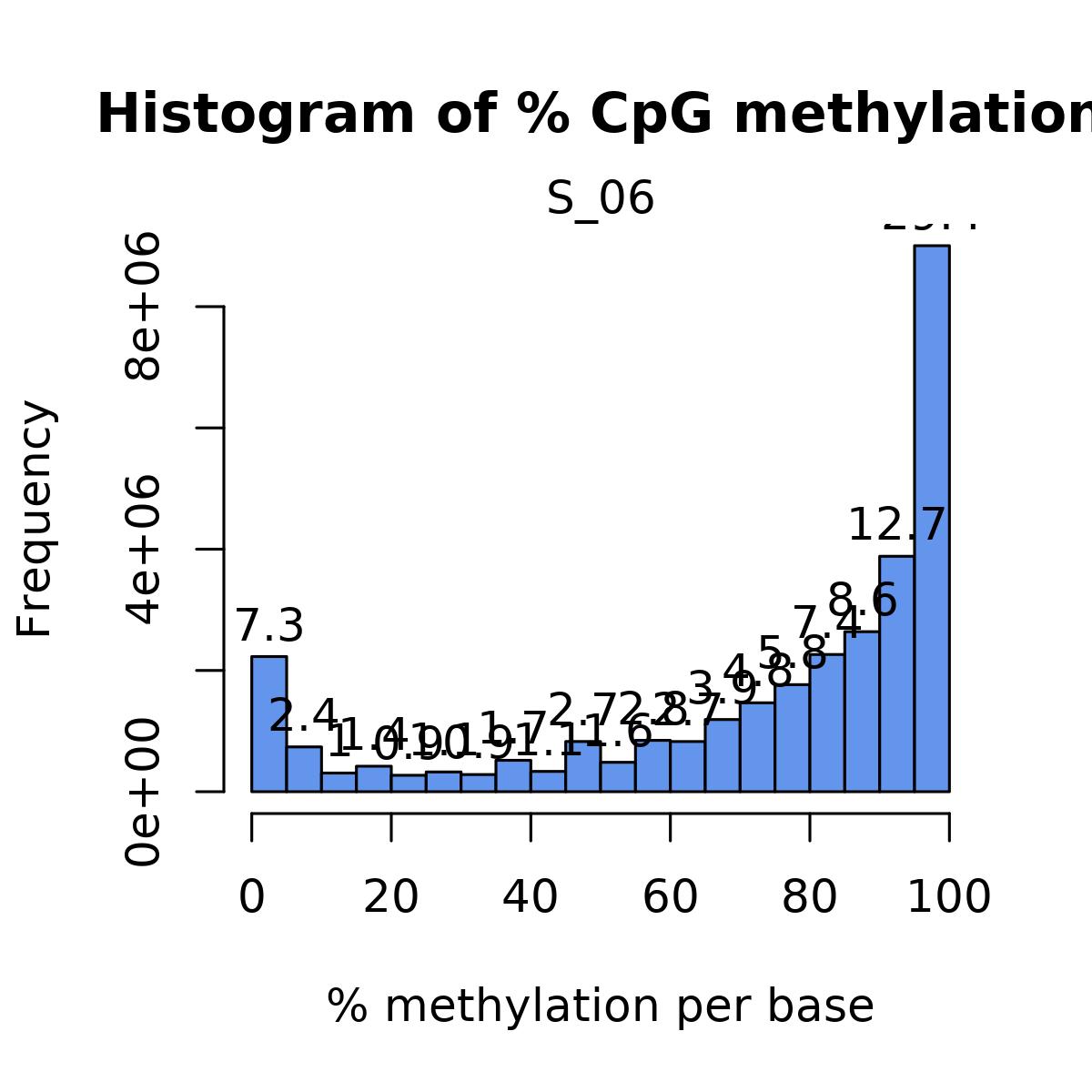

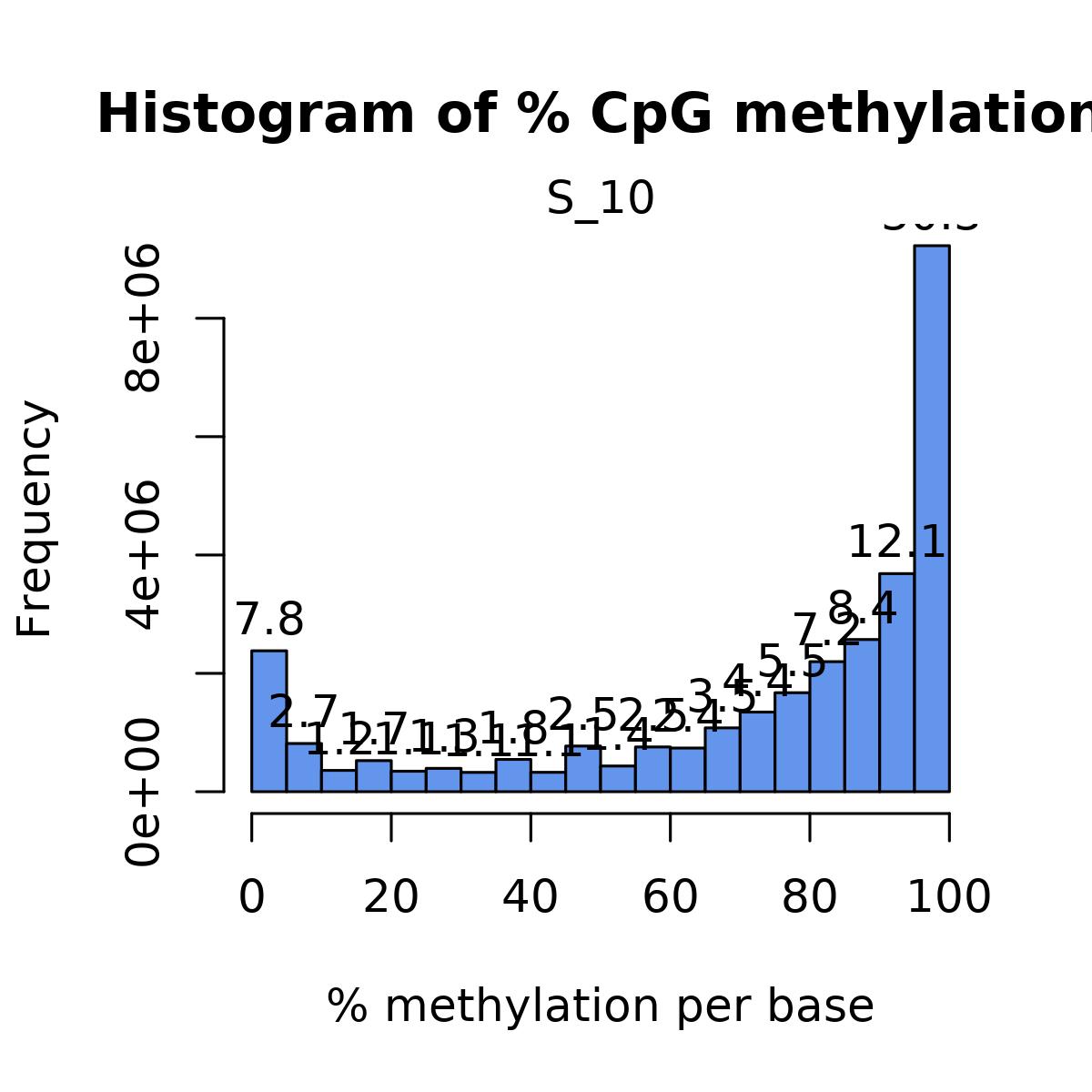

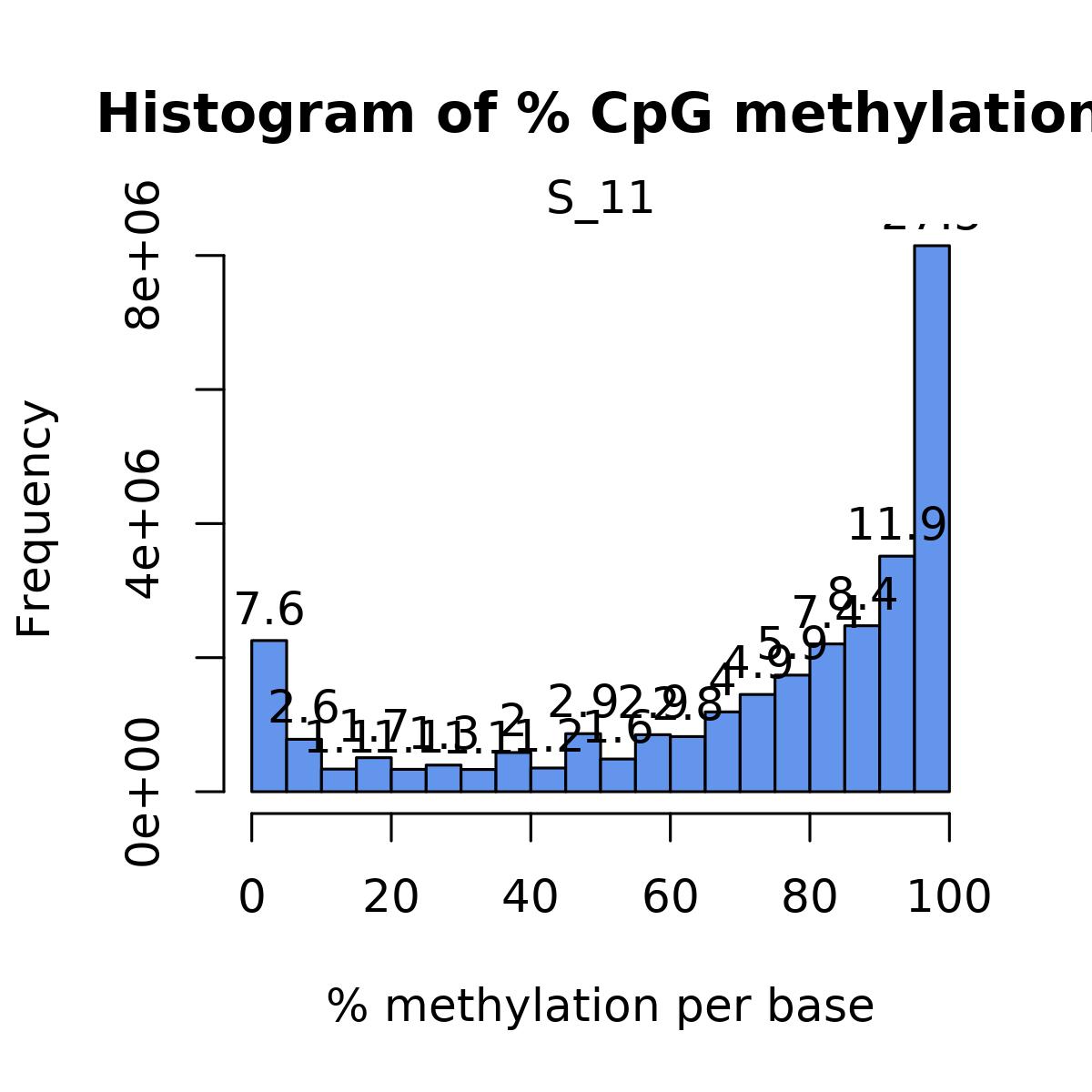

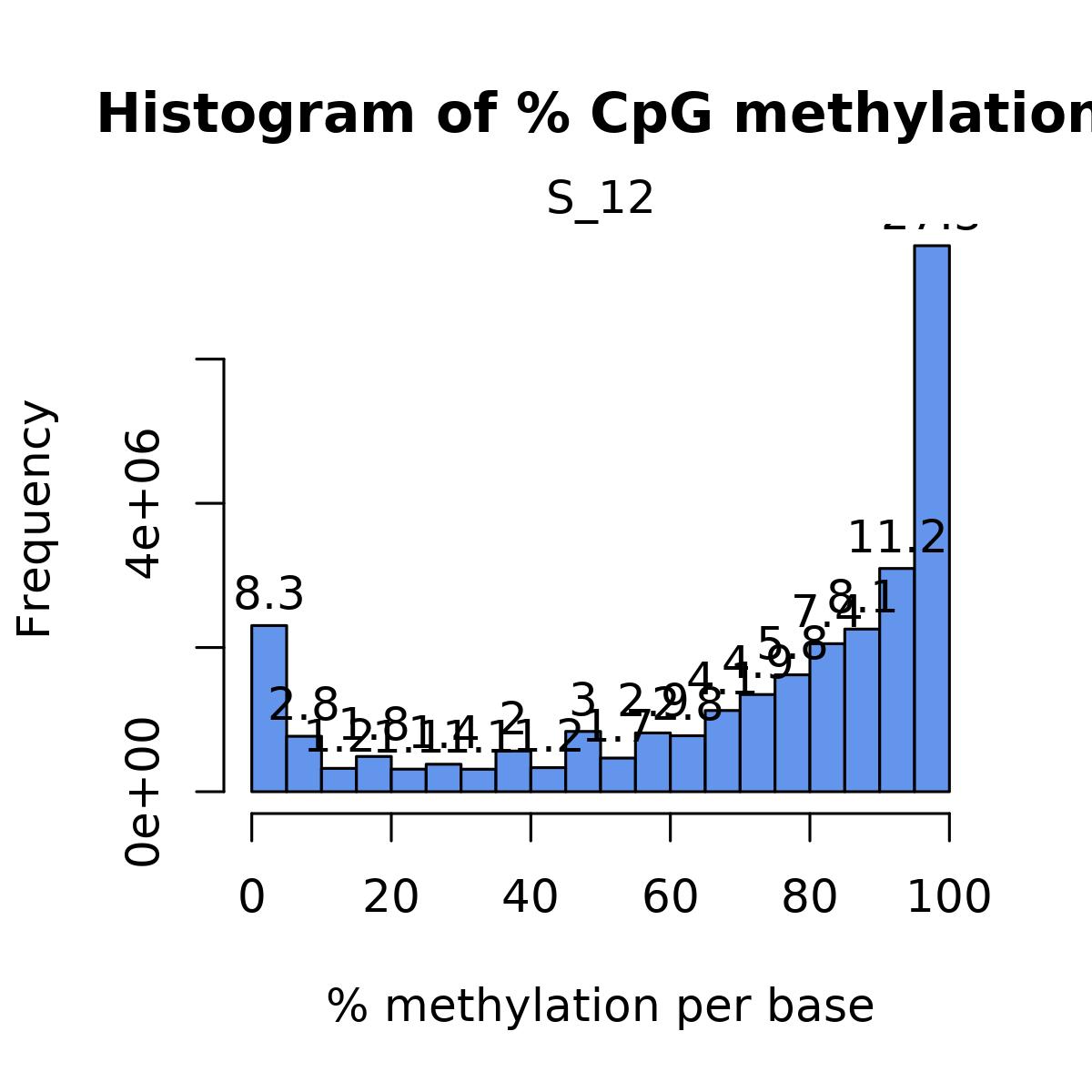

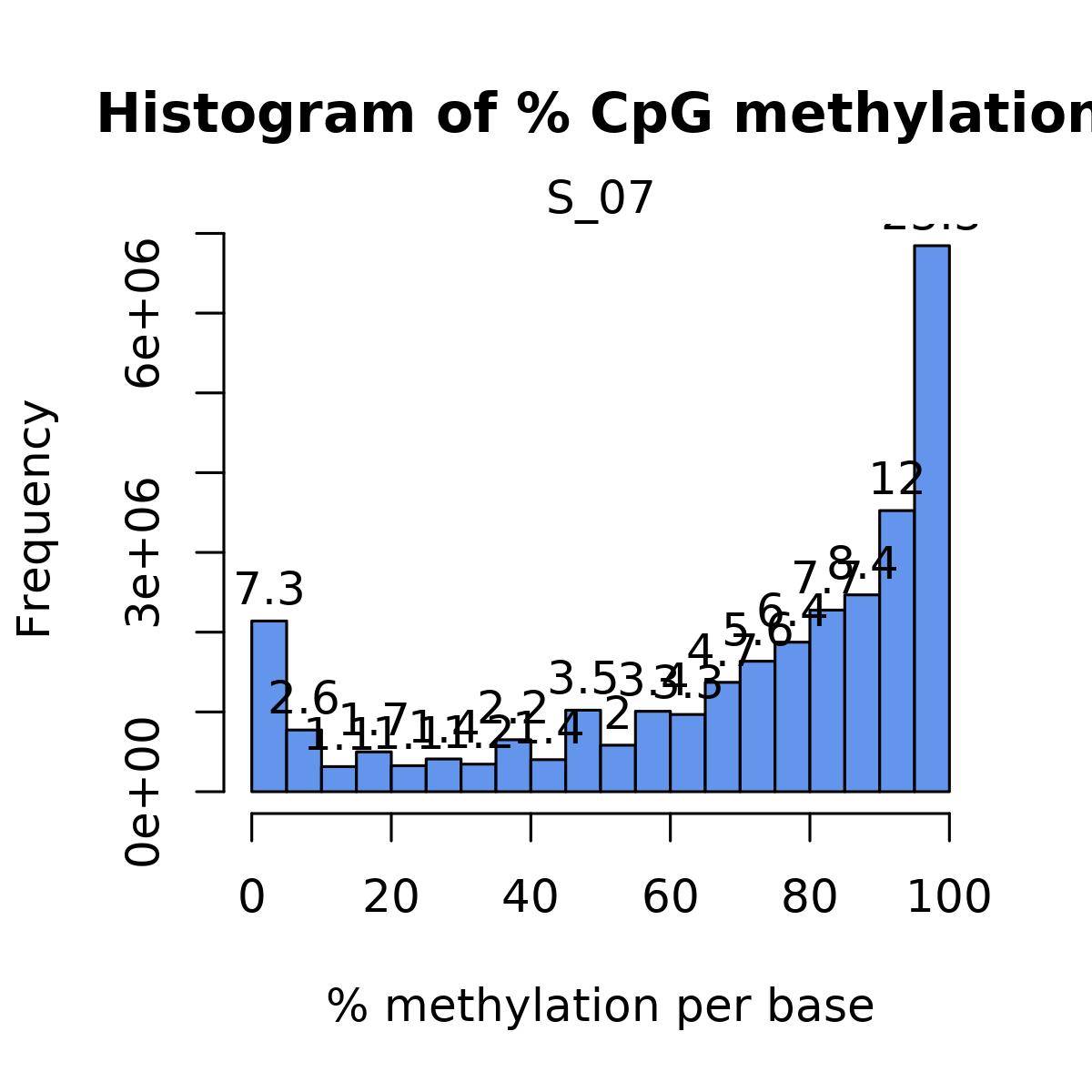

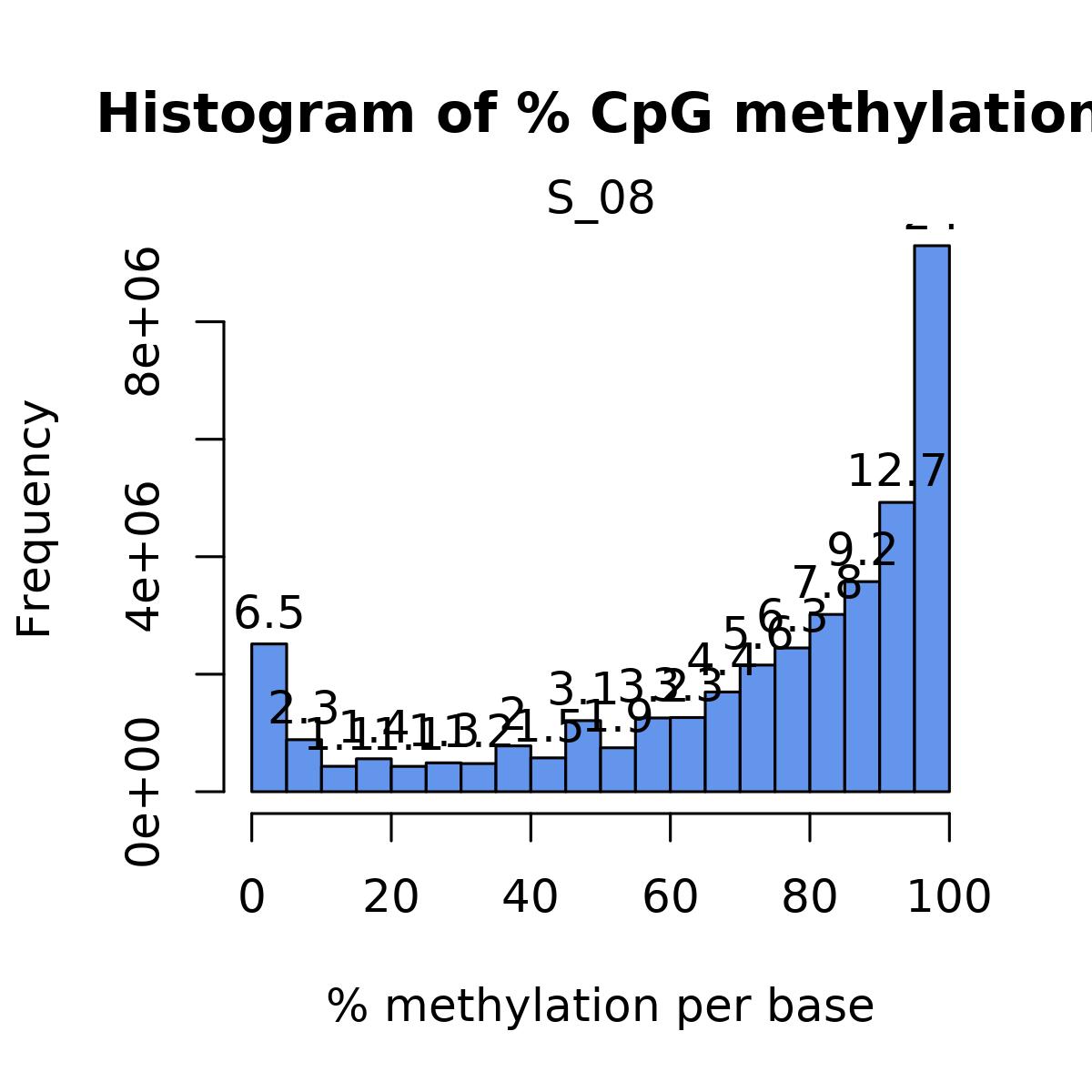

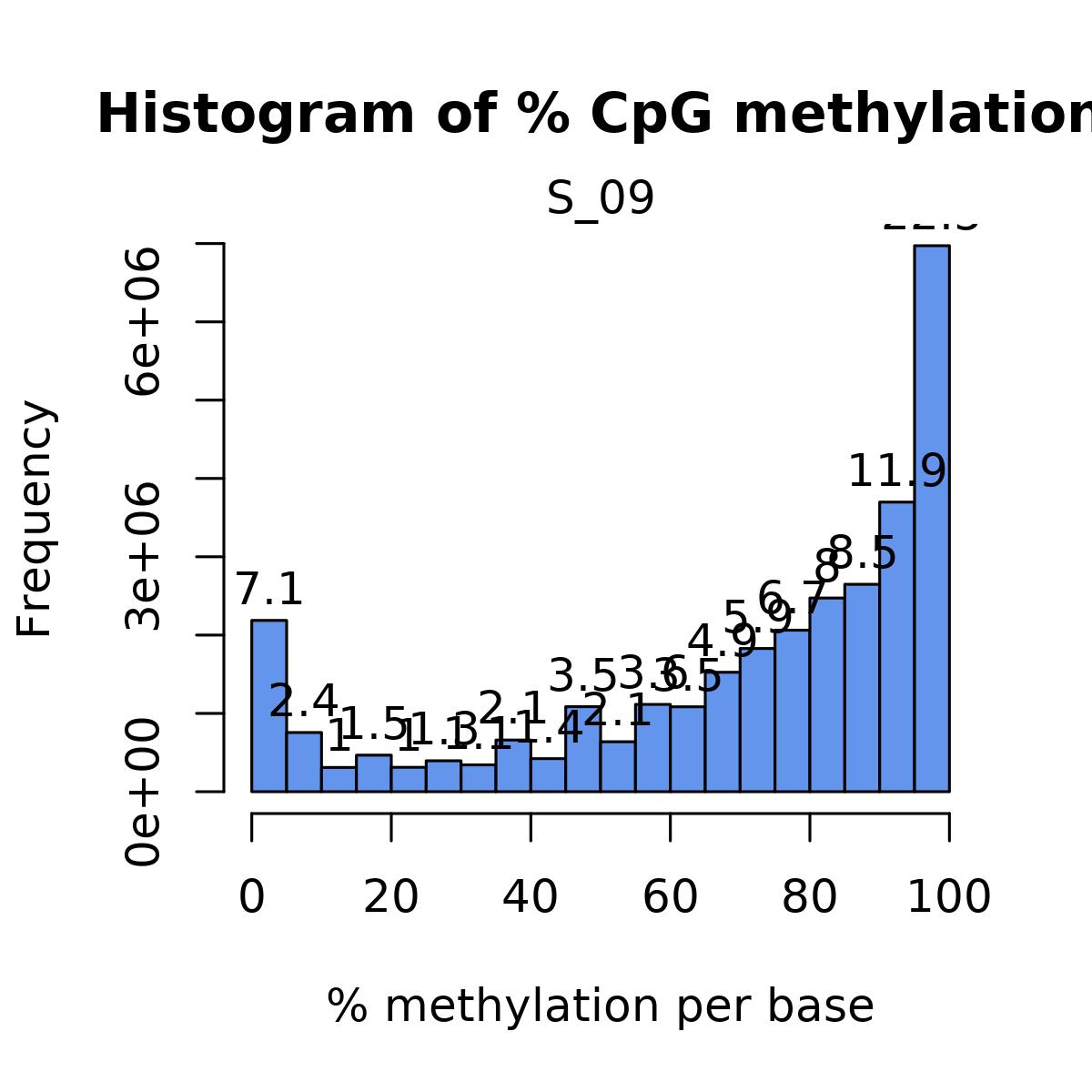


**CVID_N**

**CVID_IEL**

**Celiac**

**Control**

**Fig. S3 Histograms showing the genome-wide methylation level profile at CpG sites in the patients.** Methylation levels divided into 20 bins. A similar pattern appeared in all samples with a high peak at 95-100% and a small peak at 0-5%.

CVID: Common variable immunodeficiency; Control: healthy controls with normal duodenal biopsies; Celiac: celiac disease Marsh grade 3a, CVID_N: CVID patients with normal duodenal biopsies; CVID_IEL: CVID patients with duodenal inflammation.

**CVID_N vs Control CVID_IEL VS Control**

**Celiac_vs_Control**


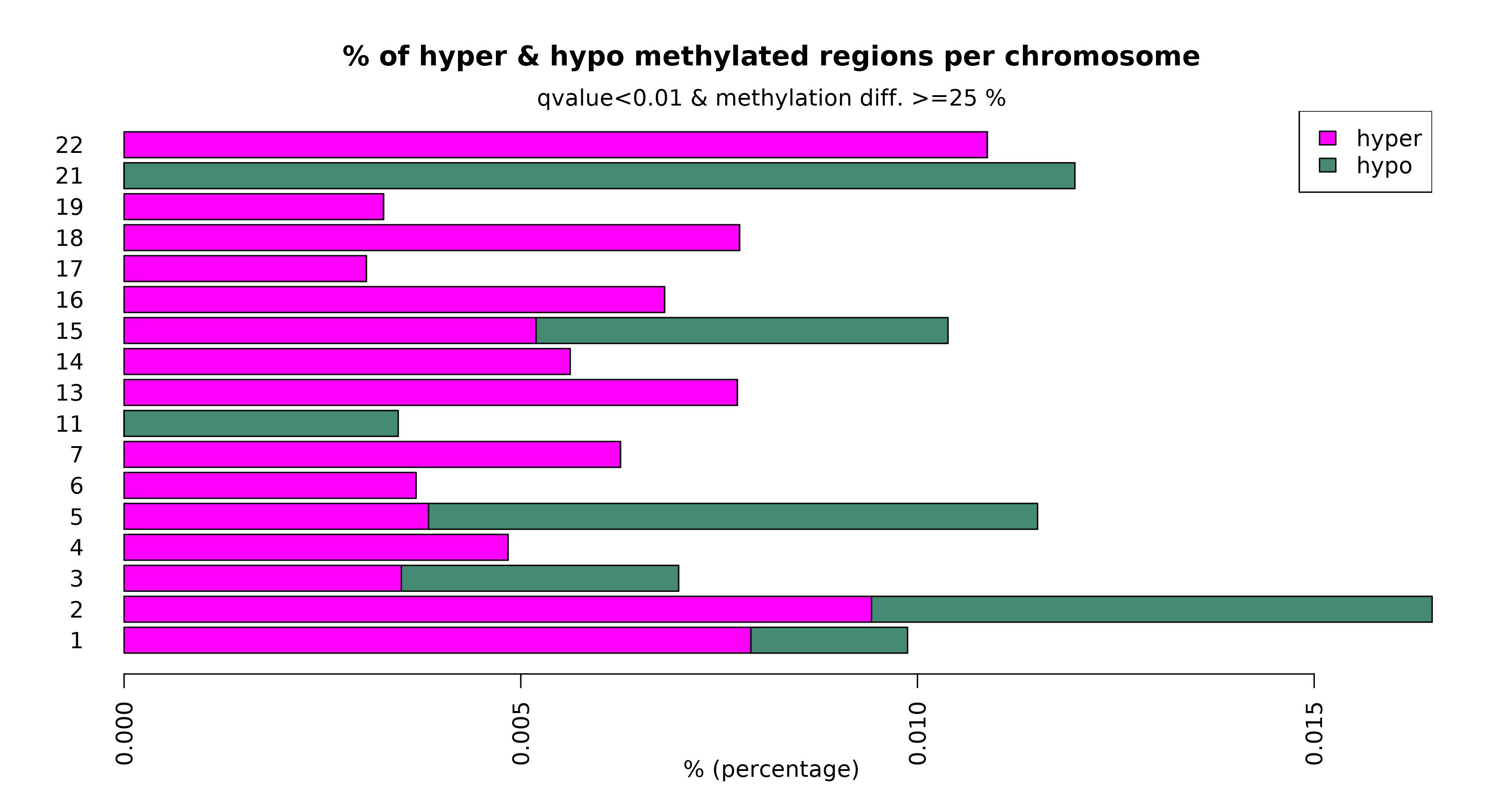

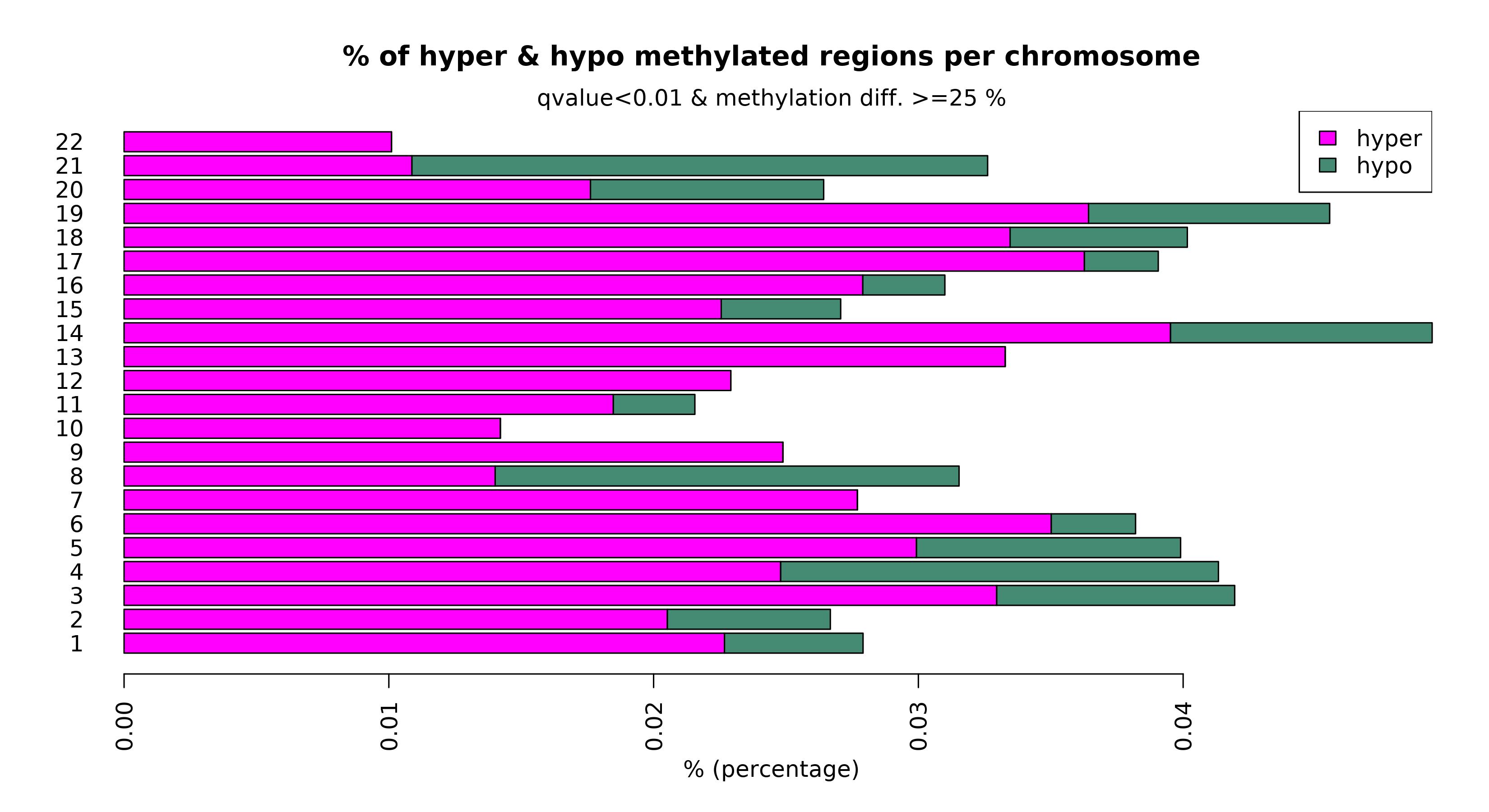

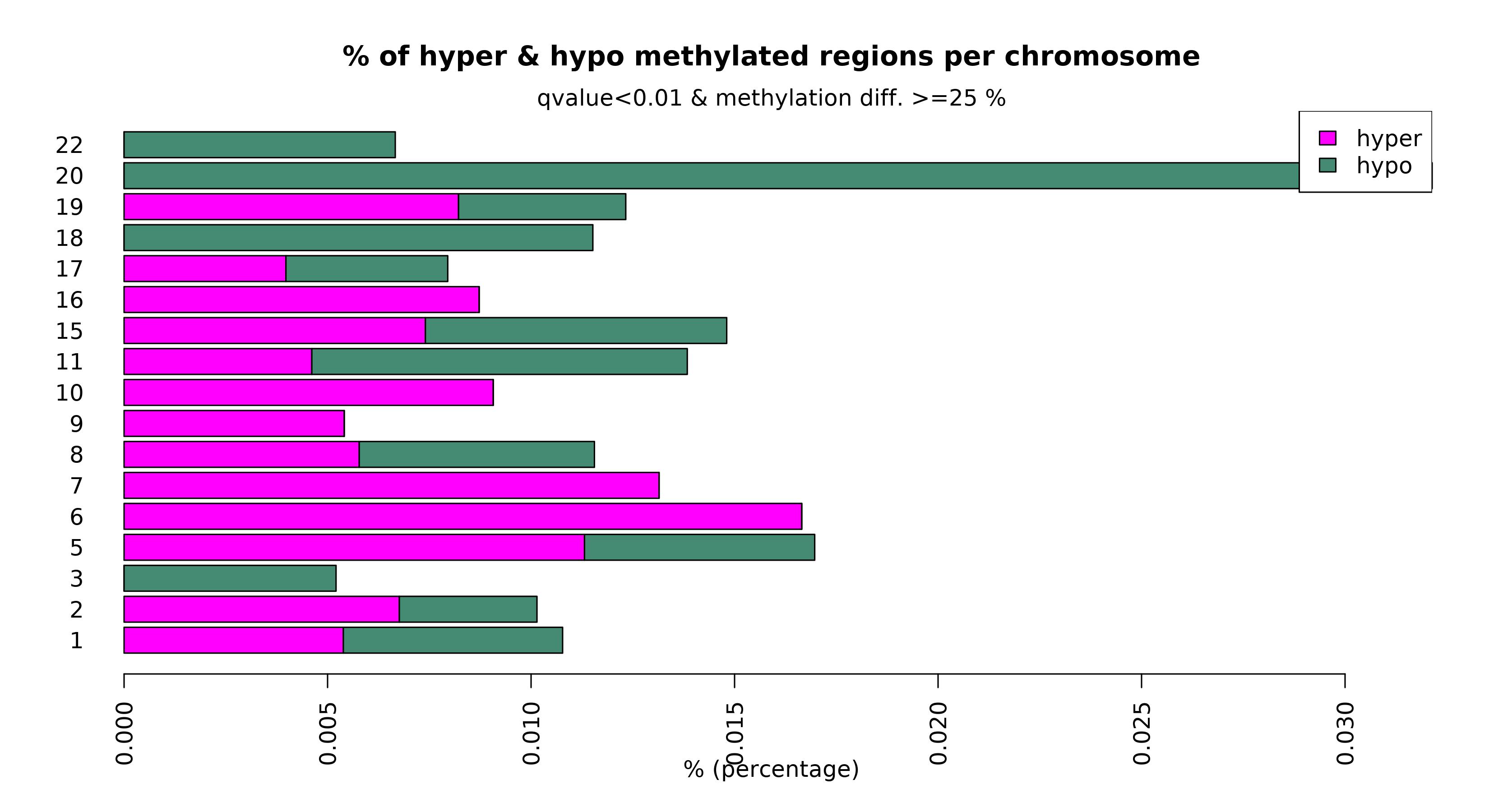


**Fig. S4 Bar plots showing the distribution of hyper- and hypomethylated regions in chromosomes**. The chromosomes without differentially methylated regions (DMRs) were excluded from Figure S4. This exclusion indicates that no DMRs were observed in those chromosomes. However, it is important to note that the absence of DMRs do not imply the absence of differential methylation within those chromosomes.

CVID: Common variable immunodeficiency; Control: healthy controls with normal duodenal biopsies; Celiac: celiac disease Marsh grade 3a, CVID_N: CVID patients with normal duodenal biopsies; CVID_IEL: CVID patients with duodenal inflammation.

**A**

**B**


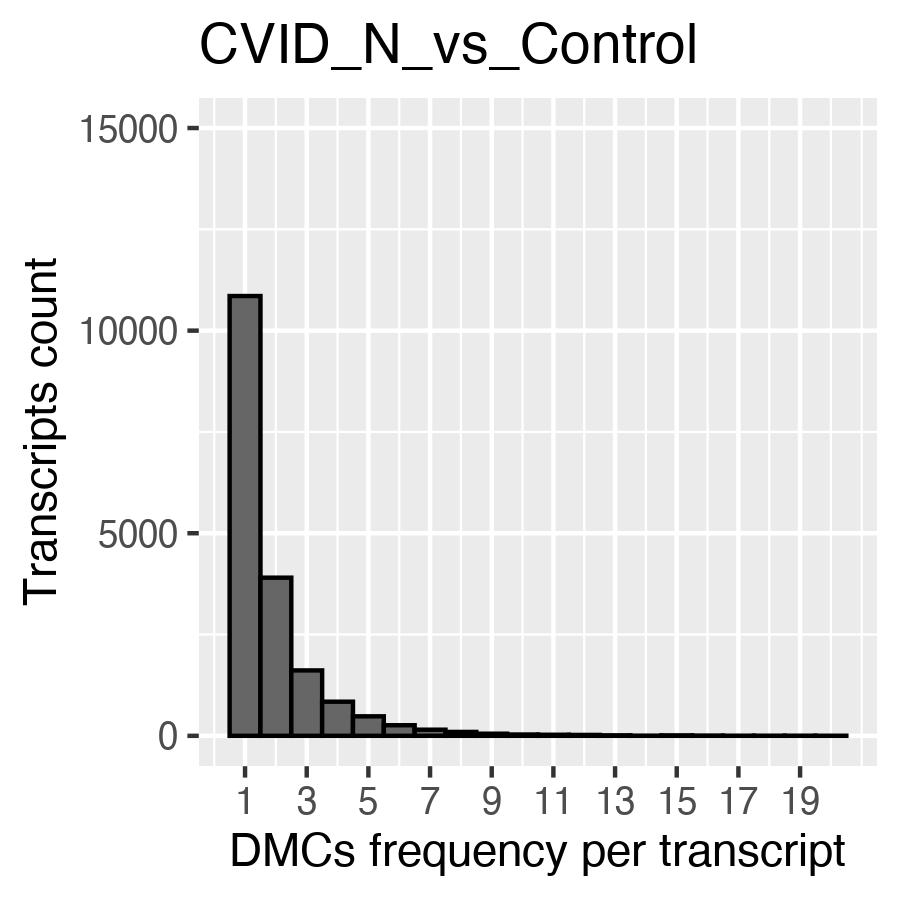

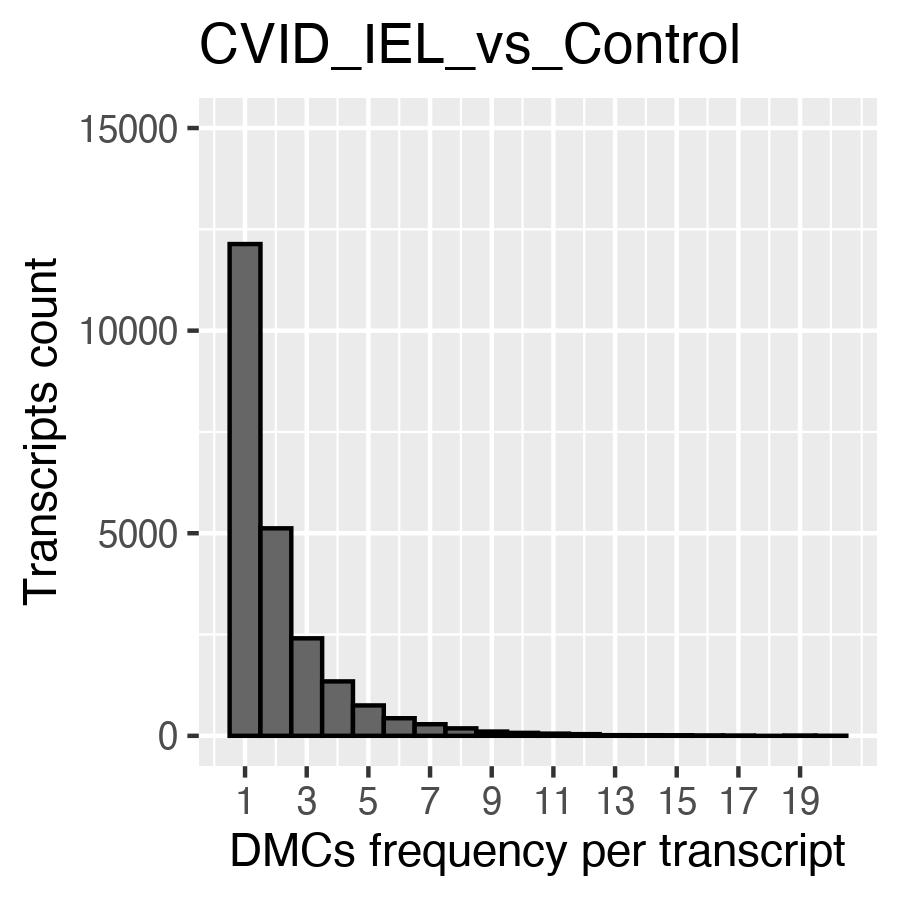

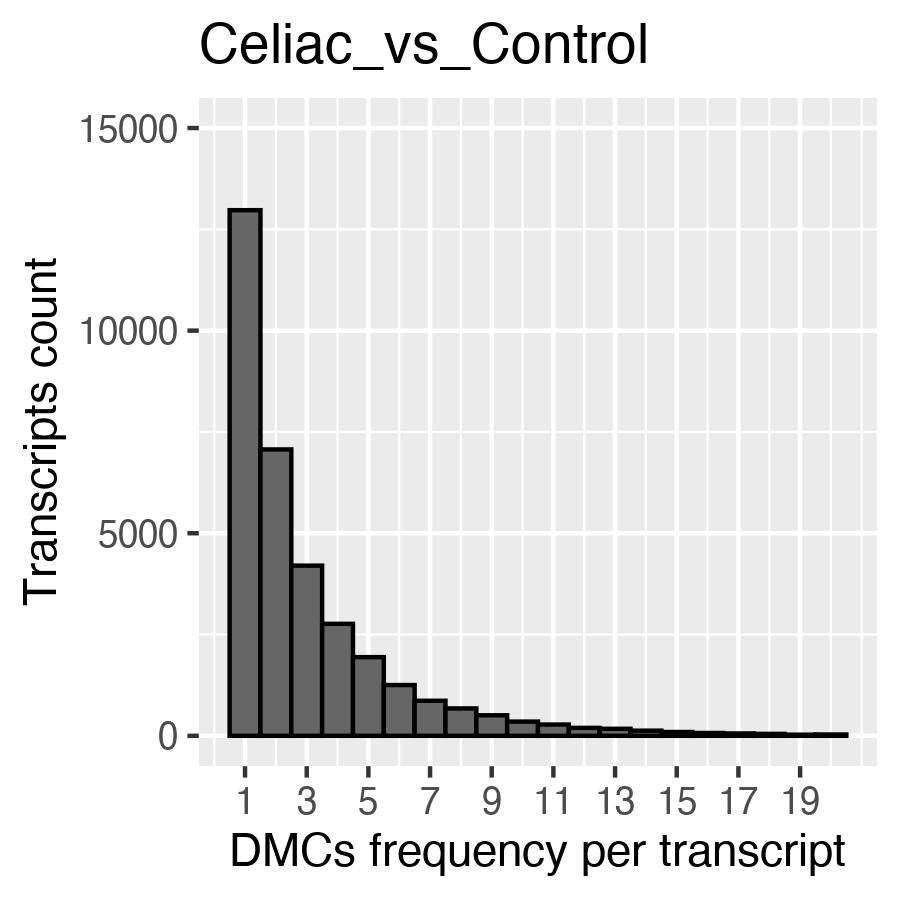

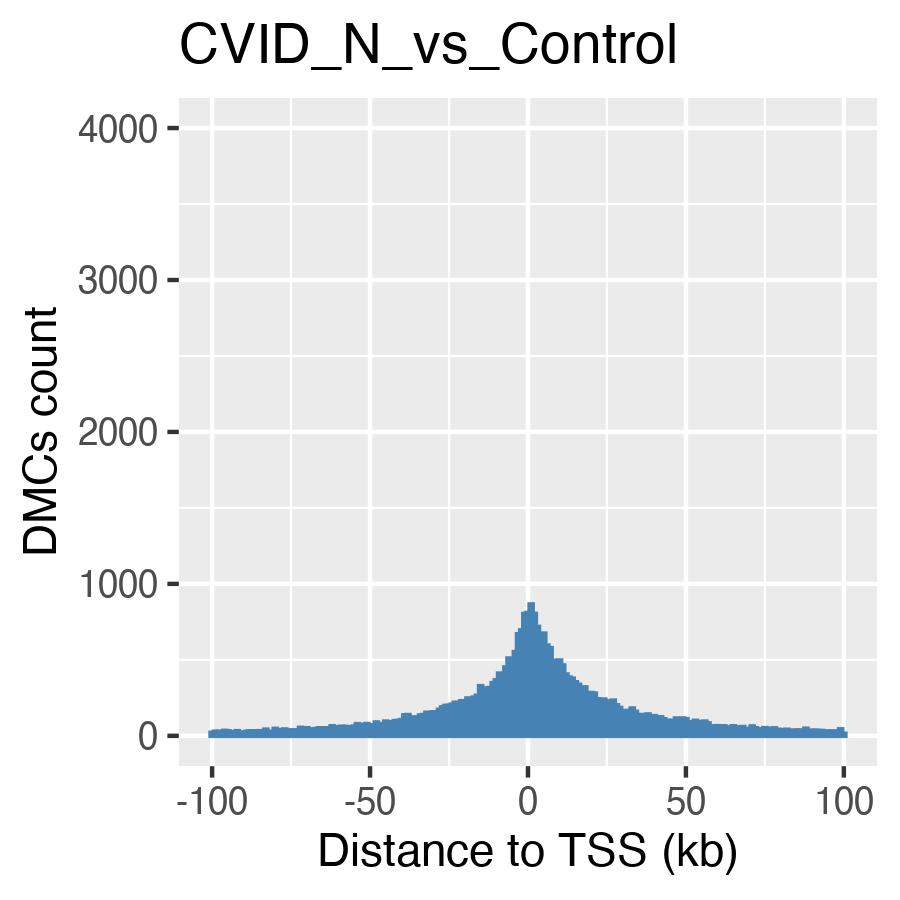

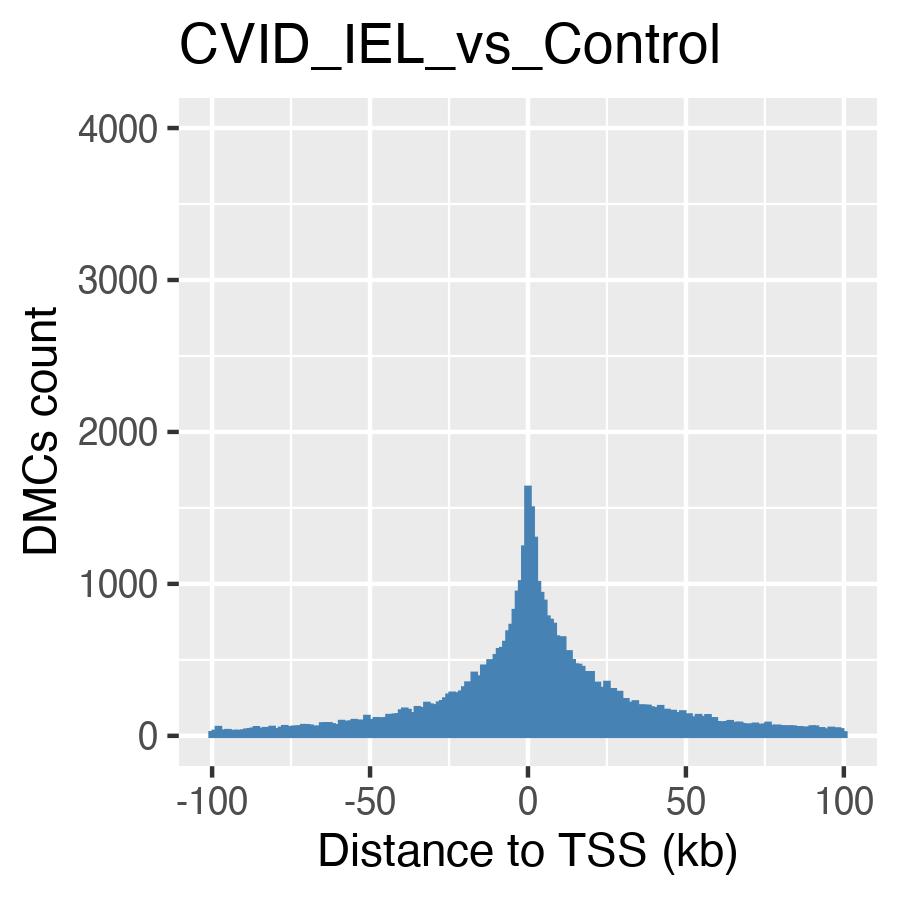

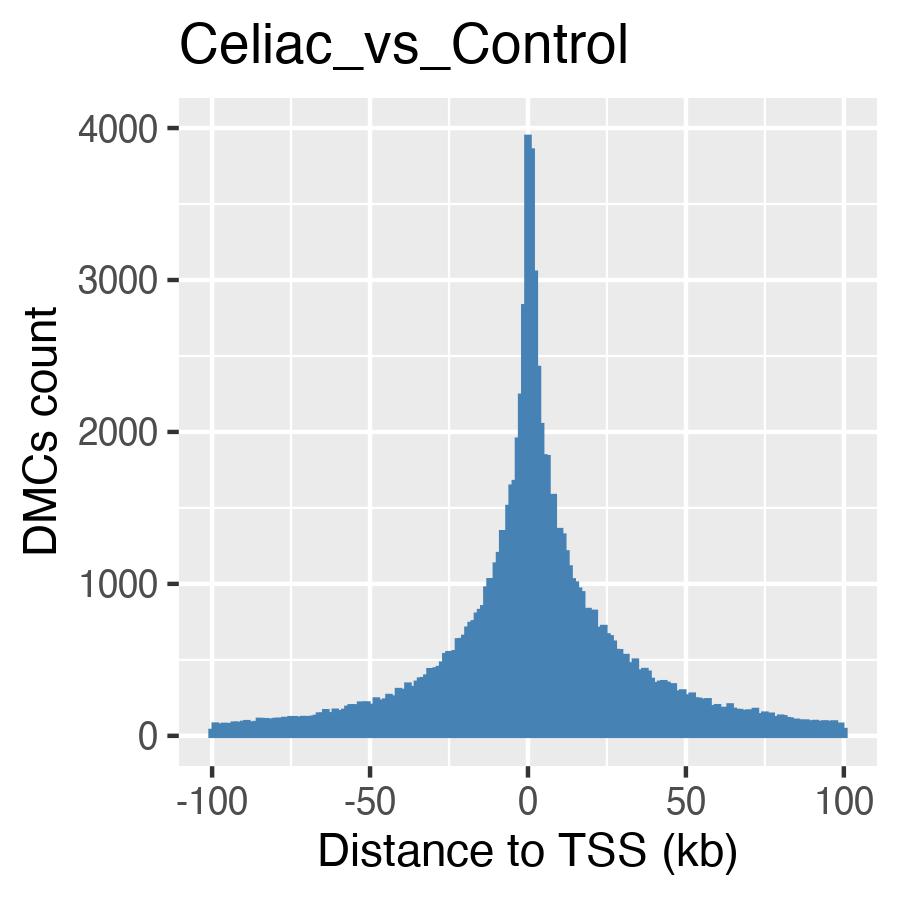


**DMC distribution**

**DMC frequency**

**Fig. S5. The distribution and frequency of differentially methylated cytosines (DMCs).** (A) Histograms showing the distribution of DMCs by distance from the TSS site, with bin size of 1 kb. (B) Bar plots displaying the number of associated transcripts for each DMC frequency. Transcripts with more than 20 DMCs are omitted.

CVID: Common variable immunodeficiency; Control: healthy controls with normal duodenal biopsies; Celiac: celiac disease Marsh grade 3a, CVID_N: CVID patients with normal duodenal biopsies; CVID_IEL: CVID patients with duodenal inflammation.


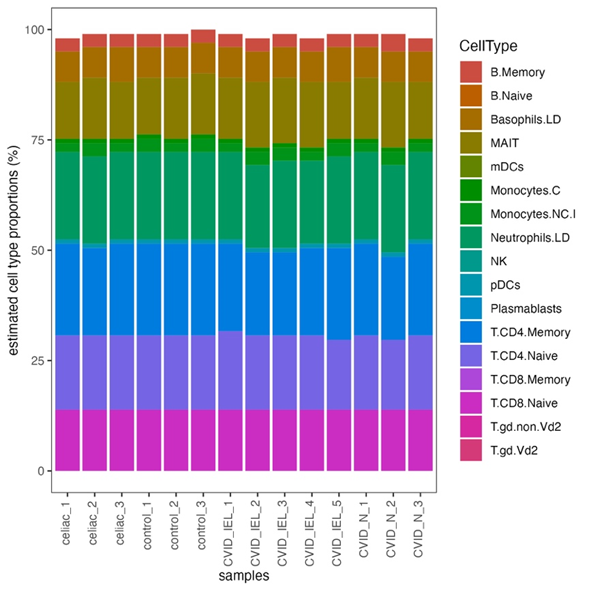


**Fig. S7.** **Immune cell proportion predicted in biopsy by deconvolution in bulk RNA-seq.** The tissue samples in RNA-seq were identical with those in GWBS data, except control_3, which was an age-matched healthy control. The number following group name in x-axis indicates sample replicates. The cell type was predicted by R package granulator, A linear model of qprogwc (quadratic programming non-negative least-squares constraints) was used in the analysis.

CVID: Common variable immunodeficiency; Control: healthy controls with normal duodenal biopsies; Celiac: celiac disease Marsh grade 3a, CVID_N: CVID patients with normal duodenal biopsies; CVID_IEL: CVID patients with duodenal inflammation.

**
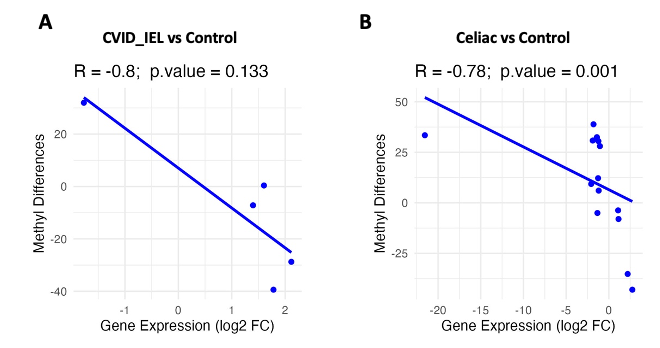
**

**Fig. S6.** **The correlation between differential methylation and gene expression.**  The common regulated genes between methylation and gene expression were extracted from GWBS and RNA-seq data. The gene expression of DEGs was presented as log2 fold changes (log2FC). The methylation of DMCs gene was presented as percentage difference. The correlation value R and p-value by a Spearman test (a rank-based association test) were indicated in the above plots. The blue dots represent common genes. The blue lines represent the smoothed linear regression between gene expression change and methylation difference. A: CVID_IEL vs Control; B: Celiac vs Control.

CVID: Common variable immunodeficiency; Control: healthy controls with normal duodenal biopsies; Celiac: celiac disease Marsh grade 3a, CVID_IEL: CVID patients with duodenal inflammation.

**References**

1 Jorgensen, S. F. *et al.* A Cross-Sectional Study of the Prevalence of Gastrointestinal Symptoms and Pathology in Patients With Common Variable Immunodeficiency. *Am. J. Gastroenterol.* **111**, 1467-1475, doi:10.1038/ajg.2016.329 (2016).

2 Ludvigsson, J. F. *et al.* Diagnosis and management of adult coeliac disease: guidelines from the British Society of Gastroenterology. *Gut* **63**, 1210-1228, doi:10.1136/gutjnl-2013-306578 (2014).

3 Rubio-Tapia, A., Hill, I. D., Kelly, C. P., Calderwood, A. H. & Murray, J. A. ACG clinical guidelines: diagnosis and management of celiac disease. *Am. J. Gastroenterol.* **108**, 656-676; quiz 677, doi:10.1038/ajg.2013.79 (2013).

4 Langmead, B. & Salzberg, S. L. Fast gapped-read alignment with Bowtie 2. *Nat Methods* **9**, 357-359, doi:10.1038/nmeth.1923 (2012).

5 Krueger, F. & Andrews, S. R. Bismark: a flexible aligner and methylation caller for Bisulfite-Seq applications. *Bioinformatics* **27**, 1571-1572, doi:10.1093/bioinformatics/btr167 (2011).

6 Akalin, A. *et al.* methylKit: a comprehensive R package for the analysis of genome-wide DNA methylation profiles. *Genome Biol* **13**, R87, doi:10.1186/gb-2012-13-10-r87 (2012).

7 Durinck, S., Spellman, P. T., Birney, E. & Huber, W. Mapping identifiers for the integration of genomic datasets with the R/Bioconductor package biomaRt. *Nat Protoc* **4**, 1184-1191, doi:10.1038/nprot.2009.97 (2009).

8 Wu, T. *et al.* clusterProfiler 4.0: A universal enrichment tool for interpreting omics data. *Innovation (Camb)* **2**, 100141, doi:10.1016/j.xinn.2021.100141 (2021).
